# Transcriptomic response to three osmotic stresses in gills of hybrid tilapia (*Oreochromis mossambicus* female × *O. urolepis hornorum* male)

**DOI:** 10.1186/s12864-020-6512-5

**Published:** 2020-01-31

**Authors:** Huanhuan Su, Dongmei Ma, Huaping Zhu, Zhigang Liu, Fengying Gao

**Affiliations:** 1 Key Laboratory of Tropical and Subtropical Fishery Resource Application and Cultivation, Ministry of Agriculture and Rural Affairs, Pearl River Fisheries Research Institute, Chinese Academy of Fishery Science, No. 1, Xingyu Road, Liwan District, Guangzhou City, 510380 China; 2 Shanghai Ocean University, College of Fisheries and Life Science, Shanghai, 201306 China

**Keywords:** Tilapia, Transcriptome, Osmoregulation, Osmotic stress

## Abstract

**Background:**

Osmotic stress is a widespread phenomenon in aquatic animal. The ability to cope with salinity stress and alkaline stress is quite important for the survival of aquatic species under natural conditions. Tilapia is an important commercial euryhaline fish species. What’s more tilapia is a good experimental material for osmotic stress regulation research, but the molecular regulation mechanism underlying different osmotic pressure of tilapia is still unexplored.

**Results:**

To elucidate the osmoregulation strategy behind its hyper salinity, alkalinity and salinity-alkalinity stress of tilapia, the transcriptomes of gills in hybrid tilapia (*Oreochromis mossambicus ♀* × *O. urolepis hornorum ♂*) under salinity stress (S: 25‰), alkalinity stress(A: 4‰) and salinity-alkalinity stress (SA: S: 15‰, A: 4‰) were sequenced using deep-sequencing platform Illumina/HiSeq-2000 and differential expression genes (DEGs) were identified. A total of 1958, 1472 and 1315 upregulated and 1824, 1940 and 1735 downregulated genes (*P*-value < 0.05) were identified in the salt stress, alkali stress and saline-alkali stress groups, respectively, compared with those in the control group. Furthermore, Kyoto Encyclopedia of Genes and Genomes pathway analyses were conducted in the significant different expression genes. In all significant DEGs, some of the typical genes involved in osmoregulation, including carbonic anhydrase (CA), calcium/calmodulin-dependent protein kinase (CaM kinase) II (CAMK2), aquaporin-1(AQP1), sodium bicarbonate cotransporter (SLC4A4/NBC1), chloride channel 2(CLCN2), sodium/potassium/chloride transporter (SLC12A2 / NKCC1) and other osmoregulation genes were also identified. RNA-seq results were validated with quantitative real-time PCR (qPCR), the 17 random selected genes showed a consistent direction in both RNA-Seq and qPCR analysis, demonstrated that the results of RNA-seq were reliable.

**Conclusions:**

The present results would be helpful to elucidate the osmoregulation mechanism of aquatic animals adapting to saline-alkali challenge. This study provides a global overview of gene expression patterns and pathways that related to osmoregulation in hybrid tilapia, and could contribute to a better understanding of the molecular regulation mechanism in different osmotic stresses.

## Background

Saline-alkali water accounts for a considerable proportion of water resources in the world. Salinity has been long recognized as one of the fundamental factors affecting aquatic species distribution and influencing physiological processes of marine and estuarine organisms, such as survival, hemolymph osmolarity, and tissue water content [1–3]. Furthermore, salinity has significant effects on physiology of aquatic organisms, and salinity adaptation is a complicated process that involves a series of physiological responses to the environment with different osmotic regulation requirements [4, 5]. Carbonate alkalinity stress was considered as a major risk factor for fishes surviving in saline-alkaline water [6, 7]. Although many studies have examined osmoregulatory physiology in freshwater and marine populations of fish [8–12], it still remains unknown about the molecular mechanism of osmotic stress tolerance [13]. Therefore, the identification and characterization of the genes and the regulatory factors involved in hyper-osmoregulation are now essential for increasing the production and the efficiency of selective breeding programs for some important fish species.

Tilapia is an important commercial fish in China, which is euryhaline fish species and it is the second most important fish after carps in aquaculture [14]. Owing to ease of aquaculture, marketability and stable market prices, the production of tilapia has quadrupled over the past decade [15], and which is promoted and farmed in more than 100 countries and regions [16]. Owing to its importance in tropical and subtropical aquaculture and its extreme euryhaline ability, tilapia has received a high level of scientific interest and is one of the most popular model species for research on fish osmoregulation [17–19]. With the increasing scarcity of freshwater available for aquaculture, tilapia that can live in brackish or hyper-osmotic water would enable expanding the range of culture and increasing the production of global tilapia [20]. In China one new hybrid tilapia strain, named Mo-Ho tilapia “Guangfu No.1”, is a hybrid from Mozambique tilapia *Oreochromis mossambicus* female × Wami tilapia *O. urolepis hornorum* male and is tolerant of hyper-osmotic water and grows well in saline pools [21]. It has been widely farmed on the southern coast of China.

With regard to ion-regulation, a set of tissues and organs such as the gills, kidney and gut plays a vital role in the teleosts [22–24]. Gill contains complex transport epithelia functions as aquatic gas exchange, acid-base regulation and excretion of nitrogenous wastes [25]. Several studies have shown that the epithelium of gill is reorganized, the distribution, number and size of chloride cells both have changed, considerably to meet the requirements of ion transport and permeability [26, 27]. When subjected to various abiotic stresses from ambient water, such as salt and alkali, gills are the first site for sensing stress signals and initiating signaling cascades at the molecular level in response to adverse environments. Through these responses, fish can regulate gene expression of regulatory, functional proteins, and morphology of gill epithelium [28–30] to enhance stress tolerance. It is important for current fish biology researches to study fish response to osmotic stress.

Recent years, with the prosperous development of the next-generation sequencing, high-throughput sequencing has been widely used to identify conserved and novel functional genes and signaling pathways in aquatic animals, such as *Litopenaeus vannamei* [13, 31], *Eriocheir sinensis* [32], *Palaemon caridean* shrimps [33] and *Mytilus trossulus* [34]. Guo et al identified three possible osmoregulation-related signaling pathways as lipid metabolism related pathways, tight junction pathway and thyroid hormone signaling pathway in Siberian sturgeon *Acipenser baeri* in response to salinity stress [35]. Lv et al showed that 552 differentially expressed genes including amino acid transport proteins and ion transport enzymes were obtained in *Portunus trituberculatus* under low salinity stress [36].

In this study, the transcriptome of the hybrid tilapia (*O. mossambicus♀* × *O. urolepis hornorum♂*) gills was sequenced by using Illumina/Hiseq-2000 RNA-seq technology for the first time. The transcriptomic data between control group and salinity challenge group, alkalinity challenge group and salinity-alkalinity challenge group were compared and analyzed to identify osmoregulation-related genes and pathways. The discoveries of this study can be conductive to illustrate the mechanism of osmotic regulation for aquatic animals adapting to salinity challenges, alkalinity challenge and salinity-alkalinity challenge.

## Results

### Sequencing and the analysis of reads

To identify mRNAs in hybrid tilapia in response to osmotic stress challenges, mRNA libraries derived from 12 groups were constructed and sequenced using the Illumina deep-sequencing platform. Using high-throughput sequencing, 54,418,529, 61,482,829, 48,353,150 and 44,867,651 average raw reads were obtained from gill tissue of control group(C), saline challenge group (S), alkali challenge group (A) and saline-alkali challenge group (SA) in hybrid tilapia, respectively (Table 1). After quality assessment, the control and challenge groups yielded 53,886,140, 60,565,527, 47,545,806 and 44,505,885 average clean reads with a Q30 average percentage 90.99, 88.63, 88.76 and 91.73%, respectively. After mapping of these clean reads and the reference genome of *Oreochromis niloticus* (https://www.ncbi.nlm.nih.gov/assembly/GCA_001858045.3), average of three sample size (*n* = 3) of four groups were 38,413,422 (71.63%), 41,942,362 (69.23%), 33,426,853 (70.45%) and 32,895,304 (73.95%) were validated as the unique matches.

**Table 1:** Number of high-throughput raw reads, clean reads and mapped clean reads generated from tilapia gills mRNA library

Sample	Number of raw reads	Q30 (%)	Number of clean reads	Q30 (%)	Mapped clean reads	Mapped ratio(%)
C1	75,721,168	88.544	74,808,692	89.44	52,233,344	69.8226
C5	43,511,812	90.96	43,136,688	91.69	30,654,690	71.0641
C6	44,022,606	91.22	43,713,040	91.85	32,352,233	74.0105
S4	55,033,428	86.48	54,047,972	87.70	37,247,738	68.9161
S5	64,567,494	86.92	63,504,896	88.06	43,706,215	68.8234
S6	64,847,564	89.3	64,143,714	90.12	44,873,133	69.9572
A4	54,989,278	85.66	53,892,592	86.98	36,759,709	68.2092
A5	45,237,800	86.22	44,285,538	87.58	30,725,853	69.3812
A6	44,832,372	90.99	44,459,288	91.71	32,794,998	73.7641
SA4	44,979,952	90.99	44,613,938	91.7	33,268,715	74.5702
SA5	46,376,590	91.01	46,009,836	91.71	33,172,654	72.099
SA6	43,246,430	91.09	42,893,882	91.78	32,244,543	75.1728

### Differential gene expression (DGE) analysis

**Fig. 1:**
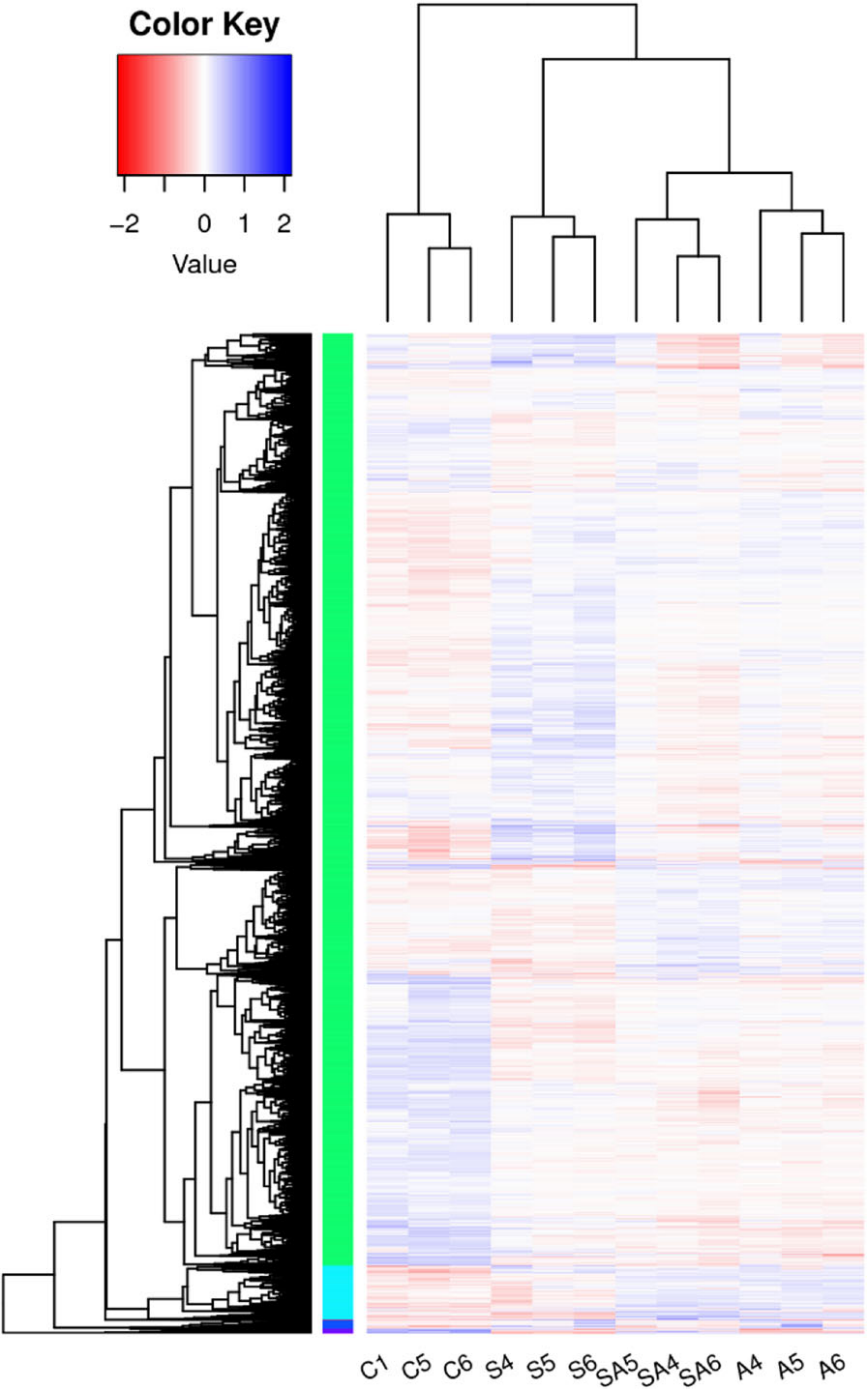
Hierarchical cluster analysis of differentially expressed genes based on the data of log ratio fold change. Heat map showing differentially expressed mRNAs upon osmotic stress in hybrid tilapia. The expression heat map was generated with the value of log_10_^FPKM^. The color scale shows the levels of differentially expressed genes: red color indicates enhanced expression of mRNA and blue color indicates decrease in expression levels of mRNA

**Fig. 2:**
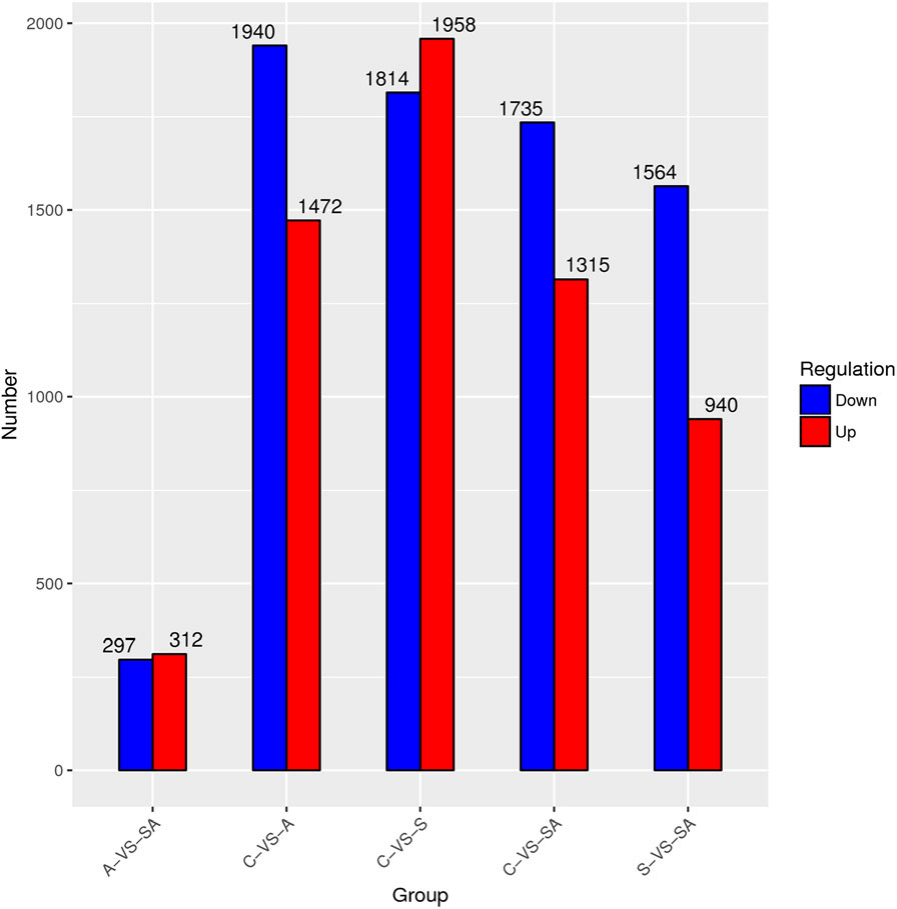
The number of differentially expressed DEGs in different groups

**Fig. 3:**
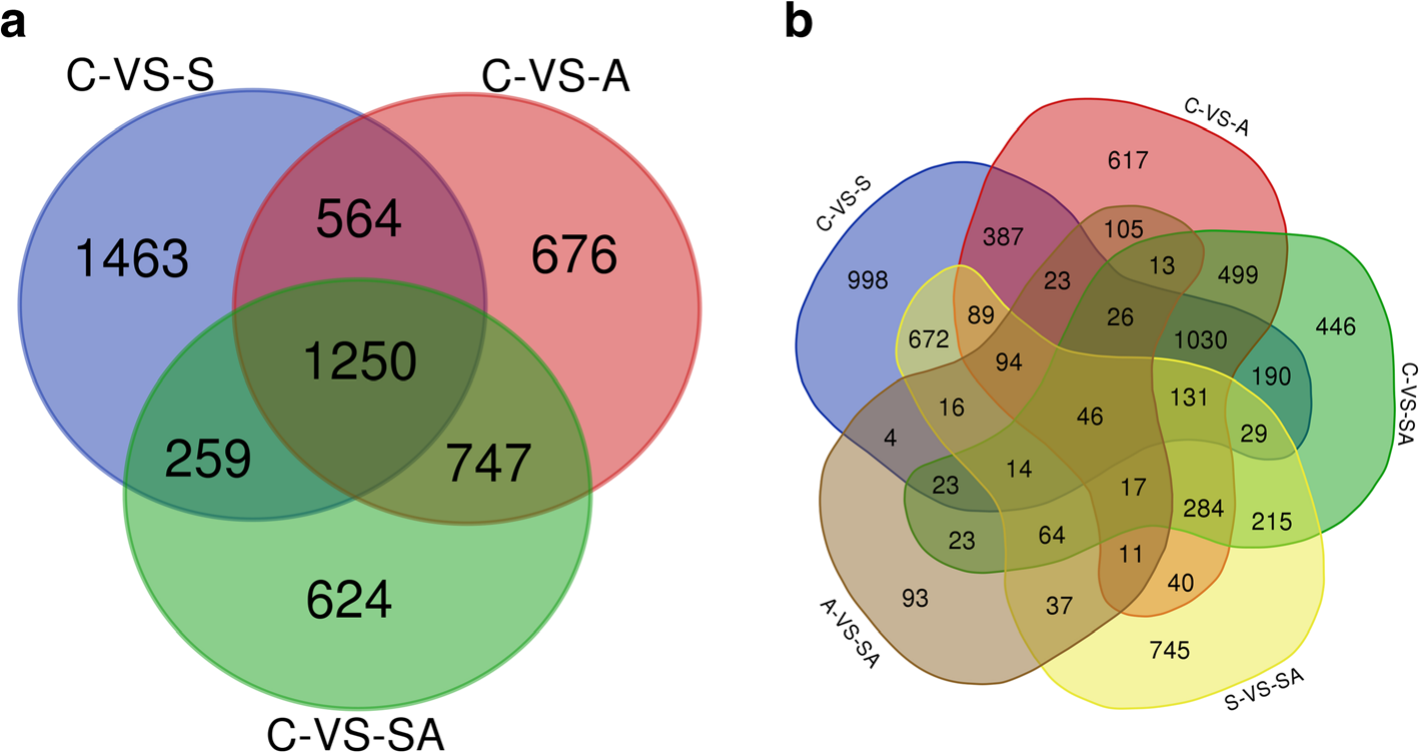
DEGs number and venn diagram of overlap of the different groups. **a**: DEGs number and venn diagram of overlap of the C vs. S, C vs. A and C vs. SA groups. **b**: DEGs number and venn diagram of overlap of the C vs. S, C vs. A, C vs. SA, S vs. SA and A vs. SA groups

Based on the expression patterns of DEGs in this study, the values of log_10_^FPKM^ (FPKM: fragments per kilo bases per million reads) were used to cluster the 12 groups, respectively, to measure and identify the similarities among the groups (Fig. 1). Based on the criteria that |logFC| > 1, *P-*value < 0.05 and FDR ≤ 0.05, the edegR and DESeq2 method were used to conducted differential expression analysis, the difference between these two methods were not significant. According to Sahraeian et al study [37], DESeq2 provided the most accurate differential analysis, so the analysis results of DESeq2 method were used to conduct the following analysis (Fig. 2). 3772 significant DEGs were detected in group S compared with control group, of which there were 1958 up-regulated genes and 1814 down-regulated genes in group S (Additional file 1: Fig. S1A). We also identified 3412 significant DEGs in group A compared with control group including 1472 up-regulated genes and 1940 down-regulated genes in group A (Additional file 1: Fig. S1B). Simultaneously, 3050 significant DEGs were detected in group SA compared with group C, of which there were 1315 up-regulated genes and 1735 down-regulated genes in group SA (Additional file 1: Fig. S1C). In addition, 2504 significant DEGs were detected in group SA compared with group S, of which there were 940 up-regulated genes and 1564 down-regulated genes in group SA (Additional file 1: Fig. S1D). Meanwhile, 609 differentially expressed genes in group SA compared with group A, which include 312 up-regulated genes and 297 down-regulated genes in group SA (Additional file 1: Fig. S1E). Further analysis indicated that a total of 1250 unigenes showed significantly different expression levels in salinity stress (S), alkalinity stress (A) and salinity-alkalinity stress (SA) (Fig. 3a). And a total of 46 unigenes showed significantly different expression levels in the C vs. S, C vs. A, C vs. SA, S vs. SA and A vs. SA groups, respectively (Fig. 3b).

### Gene ontology (GO) analysis of significant DEGs

**Fig. 4:**
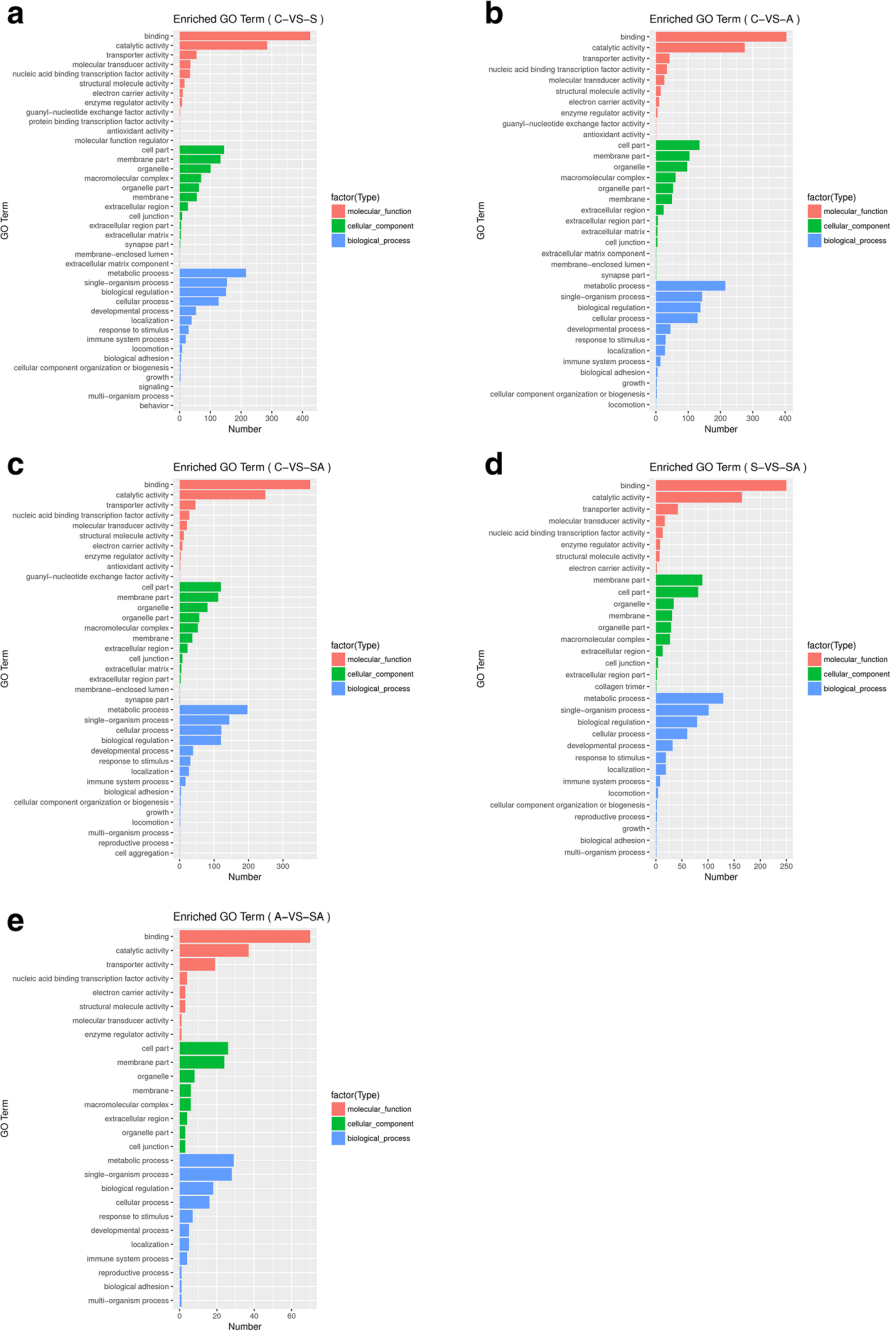
Gene Ontology (GO) classification of assembled unigenes. **a**: C vs. S; **b**: C vs. A; **c**: C vs. SA; **d**: S vs. SA; **e**: A vs. SA

In this study, GO functional analysis indicated 2300 (C vs. S), 2110 (C vs. A) and 1960 (C vs. SA) significant genes in total were classified into 40, 35 and 37 sub-categories of three major categories: biological processes, cellular components and molecular function, respectively (Fig. 4). Through the comparative analysis of GO enrichment results between different groups, it was found that some significant GO terms, such as protein binding transcription factor activity (GO:0000988), behavior (GO:0007610), signaling (GO:0023052) and molecular function regulator (GO:0098772) were enriched uniquely in salt tolerant. While in salt-alkalinity group, reproductive process (GO:0022414) and cell aggregation (GO:0098743) were uniquely enriched. We also found some significant GO terms are most likely to play an essential role in regulating osmotic stress tolerance in hybrids, such as immune system process (GO:0002376), response to stimulus (GO:0050896), transporter activity (GO:0005215) and electron carrier activity (GO:0009055). And these results of GO enrichments will provide the research clues for our following research.

### Kyoto encyclopedia of genes and genomes analysis

The top 30 KEGG pathways with the most number of annotated sequences were listed (Additional file 1: Table S1, S2, S3). These pathways consisted of cytokine-cytokine receptor interaction, protein processing in endoplasmic reticulum, autoimmune thyroid disease, pyrimidine metabolism, amino sugar and nucleotide sugar metabolism, p53 signaling pathway, biosynthesis of amino acids, glycolysis / gluconeogenesis, DNA replication, proximal tubule bicarbonate reclamation, proteasome, plycine, serine and threonine metabolism, alanine, aspartate and glutamate metabolism, TNF signaling pathway, IL-17 signaling pathway, and so on.

### Gene set enrichment analysis (GSEA) analysis of genes

To date, GSEA is being used to identify specific biological processes involved in disease outcomes and bioinformatics analysis in the medical literature and the biological research. GSEA enrichment tool was used to analysis genes between different groups, and then GO analysis was performed to find which have biological regulate function gene set. After GSEA analysis in this study, multiple GO terms were enriched in salinity group, alkalinity group and salt-alkalinity group (Additional file 1: Table S4, S5, S6) and a vast number genes were annotated to take participated in osmotic stress tolerance.

### Identification of significant DEGs and KEGG pathways related to osmoregulation

In addition, in all significant DEGs, some functional genes that related to osmoregulation, including ATP1A (sodium / potassium - transporting ATPase subunit alpha), PRLR (prolactin receptor), NKCC1 (sodium / potassium / chloride transporter), SLC9A3, NHE3 (sodium / hydrogen exchanger), TRPV4 (transient receptor potential cation channel subfamily V member 4), SLC4A2, AE2 (anion exchanger) and CLCN2 (chloride channel 2), were also identified separately. Some genes that may play important roles in osmoregulation in salinity stress, alkalinity stress and salinity-alkalinity stress were listed in Table 2.

**Table 2:** Osmoregulation-related differentially expressed genes (DEGs) regulated after stressed in group C vs. S, C vs. A and C vs. SA

Groups	Gene name	Pathway ID	Log2 (Fold Change)	*P*-value
	chloride channel 2(clcn2)	K05011	−2.57413	0.00005
	two pore calcium channel protein 3(tpcn3)	K16897	1.32543	0.00005
	transient receptor potential cation channel subfamily V member 4(TRPV4)	K04973	−1.41868	0.00005
	aquaporin-1(AQP1)	K09864	1.3813	0.00005
	aquaporin-3(AQP3)	K09876	−3.17706	0.00005
	prolactin receptor (prlr)	K05081	−1.16648	0.00005
	potassium inwardly-rectifying channel subfamily J member 2(KCNJ2)	K04996	2.21687	0.00005
	calcium/calmodulin-dependent protein kinase (CaM kinase) II (CAMK2)	K04515	1.80579	0.00005
	hyperpolarization activated cyclic nucleotide-gated potassium channel 4(HCN4)	K04957	−1.28146	0.00005
C-VS-S	phospholipid-translocating ATPase (ATP10b)	K01530	−1.29862	0.00005
	carbonic anhydrase (CA)	K01672	−1.53219	0.00005
	carbonic anhydrase 4(CA4)	K18246	3.0621	0.00105
	sodium/potassium/chloride transporter (SLC12A2/ NKCC1)	K10951	2.49218	0.00005
	solute carrier family 9 (sodium/hydrogen exchanger), member 3(SLC9A3)	K12040	−1.05992	0.00205
	Na(^+^)/H(^+^) exchange regulatory cofactor NHE-RF2(SLC9A3R2/NHERF2)	K13358	1.04245	0.00005
	sodium bicarbonate cotransporter (SLC4A4/NBC1)	K13575	−1.50471	0.00005
	mitogen-activated protein kinase 8(mapk4)	K06855	1.66571	0.00005
	mitogen-activated protein kinase 8 interacting protein 3	K04436	1.67422	0.0065
	regulator of G-protein signaling (RGS)	K16449	−3.14363	0.00005
	Solute carrier family 9 (sodium/hydrogen exchanger), member 5(NHE5)	K14723	4.08758	0.00005
	Chloride channel 2(CLCN2)	K05011	1.36324	0.00005
	Aquaporin-3(AQP3)	K09876	−1.57718	0.00005
	Aquaporin-1(AQP1)	K09864	1.27087	0.00005
	Carbonic anhydrase 4(CA4)	K18246	2.33457	0.00005
	Transient receptor potential cation channel subfamily V member 6(trpv6)	K04975	2.87291	0.00005
	Chloride channel 3/4/5(CLCN5)	K05012	−1.23945	0.00005
	Two pore calcium channel protein 3(TPCN3)	0.00005	1.12829	0.00005
	Potassium inwardly-rectifying channel subfamily J member 2(KCNJ2)	K04996	1.73207	0.00005
	Hyperpolarization activated cyclic nucleotide-gated potassium channel 4(HCN4)	K04957	−1.18463	0.00005
	Potassium channel subfamily K member 5(KCNK5)	K04916	1.31934	0.00005
C-VS-A	Calcium/calmodulin-dependent protein kinase (cam kinase) II (CAMK2)	K04515	−1.1669	0.004
	Phospholipid-translocating atpase (ATP10b)	K01530	−1.11012	0.00005
	Phospholipid-translocating atpase (ATP8b4)	K01530	1.25176	0.00005
	Sodium/potassium-transporting atpase subunit alpha (ATP1A)	K01539	−1.98927	0.00005
	Solute carrier family 8 (sodium/calcium exchanger)(SLC8A1)	K05849	3.17412	0.00005
	Solute carrier family 4 (anion exchanger), member 2(SLC4A2)	K13855	−2.43824	0.00005
	Sodium bicarbonate cotransporter (SLC4A4/NBC1)	K13575	1.30546	0.00005
	Sodium/potassium/chloride transporterslc12a2/NKCC1)	K10951	1.05921	0.00005
	MFS transporter, SP family, solute carrier family 2 member 8(SLC2A8)	K08145	1.82715	0.01315
	Mitogen-activated protein kinase6(MAPK6)	K06855	1.12481	0.00005
	Mitogen-activated protein kinase4 kinase 4(MAPK4k4)	K04407	−1.96545	0.00005
	Mitogen-activated protein kinase 8 interacting protein 3	K04436	1.0482	0.00025
	Regulator of G-protein signaling (rgs12)	K16449	1.23556	0.01035
	Carbonic anhydrase (CA)	K01672	−1.63577	0.00005
	Carbonic anhydrase 4(CA4)	K18246	2.00696	0.00005
	Potassium channel subfamily K member 5(kcnk5)	K04916	1.86814	0.00005
	Transient receptor potential cation channel subfamily V member 6(TRPV6)	K04975	4.23057	0.00005
	Calcium/calmodulin-dependent protein kinase (cam kinase) II (CAMK2)	K04515	1.29023	0.00005
	Chloride channel 3/4/5(CLCN5)	K05012	1.20394	0.00005
	Hyperpolarization activated cyclic nucleotide-gated potassium channel 4(HCN4)	K04957	−1.36233	0.00005
C-VS-SA	Phospholipid-translocating atpase (ATP10b)	K01530	−2.14108	0.0008
	Aquaporin-9(AQP9)	K09877	2.47715	0.0046
	Aquaporin-3(AQP3)	K09876	−2.15244	0.00005
	Adenylate cyclase 3(ADCY3)	K08043	1.52349	0.0108
	Sodium/potassium/chloride transporter (SLC12A2/NKCC1)	K10951	1.21719	0.00005
	Sodium bicarbonate cotransporter (SLC4A4/NBC1)	K13575	1.23287	0.00005
	Solute carrier family 8 (sodium/calcium exchanger)(SLC8A1)	K05849	4.74133	0.00005
	Mitogen-activated protein kinase4(MAPK4)	K06855	1.36517	0.00005
	Mitogen-activated protein kinase4 kinase 4(MAPKk4)	K04407	1.43318	0.0017
	Regulator of G-protein signaling (RGS)	K16449	−1.93502	0.00005

A comparative analysis of the KEGG pathways enriched in the three groups revealed that 25 of the pathways were significantly enriched in all the three groups, including steroid biosynthesis (ko00100), IL-17 signaling pathway (ko04657), glycine, serine and threonine metabolism (ko00260), Cell cycle (ko04110), and so on. However, only two pathways, proteasome (ko03050) and the p53 signaling pathway (ko04115), were enriched in the salinity group and the alkalinity group. Meanwhile, three pathways, fructose and mannose metabolism (ko00051), glycolysis / gluconeogenesis (ko00010) and alanine, aspartate and glutamate metabolism (ko00250), were enriched in the salinity and saline-alkali group. While in alkali stress group and salinity-alkalinity group, there were 18 pathways were significantly enriched, include natural killer cell mediated cytotoxicity (ko04650), synthesis and degradation of ketone bodies (ko00072), glycosaminoglycan degradation (ko00531), and so on (Table 3).

**Table 3:** Comparative analysis of the KEGG pathways enriched in the three groups

Group	Total	Payhway ID	Pathway description
S & A & SA	25	ko00100	Steroid biosynthesis
		ko05332	Graft-versus-host disease
		ko04657	IL-17 signaling pathway
		ko00512	Mucin type O-glycan biosynthesis
		ko05133	Pertussis
		ko05330	Allograft rejection
		ko05320	Autoimmune thyroid disease
		ko00660	C5-Branched dibasic acid metabolism
		ko00520	Amino sugar and nucleotide sugar metabolism
		ko05322	Systemic lupus erythematosus
		ko00260	Glycine, serine and threonine metabolism
		ko03430	Mismatch repair
		ko04111	Cell cycle - yeast
		ko01200	Carbon metabolism
		ko01230	Biosynthesis of amino acids
		ko00909	Sesquiterpenoid and triterpenoid biosynthesis
		ko00680	Methane metabolism
		ko04110	Cell cycle
		ko03030	DNA replication
		ko04940	Type I diabetes mellitus
		ko04612	Antigen processing and presentation
		ko00052	Galactose metabolism
		ko04964	Proximal tubule bicarbonate reclamation
		ko00643	Styrene degradation
		ko00240	Pyrimidine metabolism
A & S	2	ko03050	Proteasome
		ko04115	p53 signaling pathway
S & SA	3	ko00051	Fructose and mannose metabolism
		ko00010	Glycolysis / Gluconeogenesis
		ko00250	Alanine, aspartate and glutamate metabolism
A & SA	18	ko04650	Natural killer cell mediated cytotoxicity
		ko00072	Synthesis and degradation of ketone bodies
		ko05166	HTLV-I infection
		ko04113	Meiosis - yeast
		ko00230	Purine metabolism
		ko00531	Glycosaminoglycan degradation
		ko02026	Biofilm formation - *Escherichia coli*
		ko04145	Phagosome
		ko05203	Viral carcinogenesis
		ko05169	Epstein-Barr virus infection
		ko03420	Nucleotide excision repair
		ko01524	Platinum drug resistance
		ko00480	Glutathione metabolism
		ko05416	Viral myocarditis
		ko05168	Herpes simplex infection
		ko03460	Fanconi anemia pathway
		ko03440	Homologous recombination
		ko05130	Pathogenic Escherichia coli infection
S	9	ko05310	Asthma
		ko00710	Carbon fixation in photosynthetic organisms
		ko04060	Cytokine-cytokine receptor interaction
		ko04512	ECM-receptor interaction
		ko00720	Carbon 2fixation pathways in prokaryotes
		ko00410	beta-Alanine metabolism
		ko04141	Protein processing in endoplasmic reticulum
		ko00910	Nitrogen metabolism
		ko00590	Arachidonic acid metabolism
A	6	ko00511	Other glycan degradation
		ko00513	Various types of N-glycan biosynthesis
		ko04668	TNF signaling pathway
		ko05164	Influenza A
		ko00980	Metabolism of xenobiotics by cytochrome P450
		ko00650	Butanoate metabolism
SA	12	ko04978	Mineral absorption
		ko00640metabolism	Propanoate
		ko02020	Two-component system
		ko00630	Glyoxylate and dicarboxylate metabolism
		ko05144	Malaria
		ko04514	Cell adhesion molecules (CAMs)
		ko00363	Bisphenol degradation
		ko05410	Hypertrophic cardiomyopathy (HCM)
		ko00521	Streptomycin biosynthesis
		ko00290	Valine, leucine and isoleucine biosynthesis
		ko00500	Starch and sucrose metabolism
		ko00770	Pantothenate and CoA biosynthesis

In the present study, 17 osmoregulation-related signaling pathways as steroid biosynthesis (ko00100), mucin type O-glycan biosynthesis (ko00512), cell cycle (ko04110), proximal tubule bicarbonate reclamation (ko04964), glycine, serine and threonine metabolism (ko00260), alanine, aspartate and glutamate metabolism (ko00250), p53 signaling pathway (ko04115), glycolysis / gluconeogenesis (ko00010), glutathione metabolism (ko00480), ECM-receptor interaction (ko04512), protein processing in endoplasmic reticulum (ko04141), arachidonic acid metabolism (ko00590), TNF signaling pathway (ko04668), mineral absorption (ko04978), cell adhesion molecules (CAMs) (ko04514), valine, leucine and isoleucine biosynthesis (ko00290), pantothenate and CoA biosynthesis (ko00770) were showed in Table 3.

### Significant DEGs analysis in different groups

Hierarchical cluster, GO function analysis and KEGG pathway analysis were carried out for significant DEGs in each interval in Venn chart (Fig. 3a). Hierarchical clusters were analyzed of significant DEGs that were expression uniquely in salinity stress (S), alkalinity stress (A), salinity-alkalinity stress (SA), salinity stress and alkalinity stress (S & A), salinity stress and salinity-alkalinity stress (S & SA), alkalinity stress and salinity-alkalinity stress (A & SA), the heat map of significant DEGs were showed in Additional file 1: Fig. S2.

**Fig. 5:**
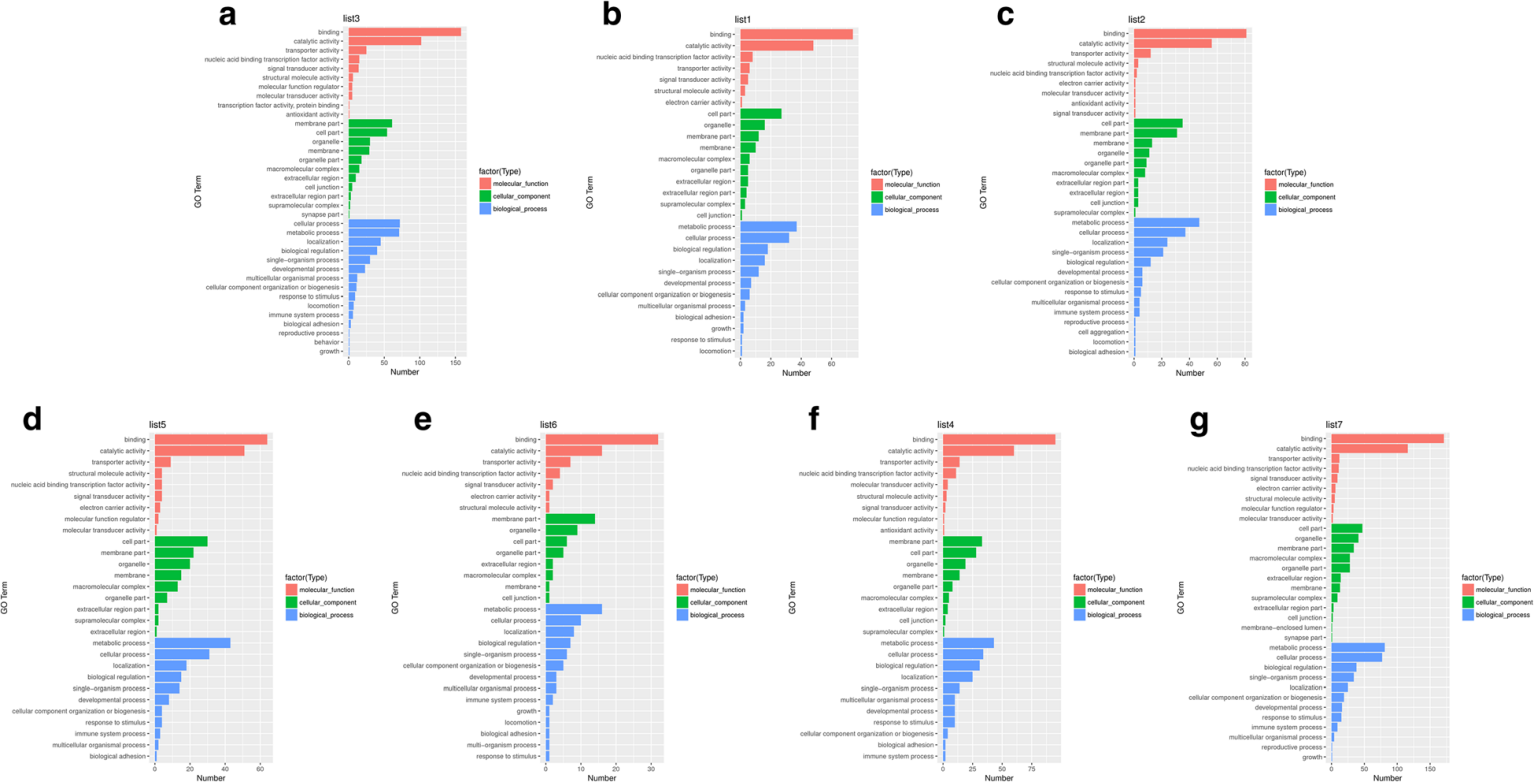
Gene Ontology (GO) classification of differential expression genes in group S, A, SA, S & A, S & SA, A & SA S, and A & SA. **a**: S; **b**: A; **c**: SA; **d**: S & A; **e**: S & SA; **f**: A & SA; **g**: S, A & SA

Furthermore, GO analyses were performed to identify similarities and differences among differentially expressed genes only in salinity stress (S), alkalinity stress (A), salinity-alkalinity stress (SA), alkalinity and salinity-alkalinity stress (A, SA), salinity and salinity-alkalinity stress (S, SA) and three osmotic stress shared (S, A, SA). By comparing the analysis results of GO enrichments, we found that transcription factor activity, protein binding (GO:0000988) and behavior (GO:0007610) were uniquely enriched in salinity stress (A). We also found that cell aggregation (GO:0098743), multi-organism process (GO:0051704) and membrane-enclosed lumen (GO:0031974) were uniquely enriched in salinity-alkalinity stress (SA), salinity-alkalinity stress (S, SA) and three osmotic stress (S, A, SA), respectively. All these analysis results will provide research data and clues for the future research directions, and are essential for the research of the molecular mechanism of osmotic regulation in teleost. The results of GO enrichment were listed as Fig. 5.

Meanwhile, KEGG analyses were also performed to identify similarities and differences among differentially expressed genes uniquely in salinity stress (S), alkalinity stress (A), salinity-alkalinity stress (SA), alkalinity and salinity-alkalinity stress (A & SA), salinity and salinity-alkalinity stress (S & SA) and three osmotic stress shared (S & A & SA) groups. A total of 133, 226 and 58 significant DEGs of only expressed in S, A and SA were annotated in KEGG and grouped into 12, 17 and 13 pathway terms, respectively (Tables 4, 5, and 6). Furthermore, 15, 12, 27 and 29 pathway terms were annotated in S & A, S & SA, A & SA and S & A & SA groups, respectively (Tables 7, 8, 9, and 10). We found that cytokine-cytokine receptor interaction, ECM-receptor interaction, glycolysis / gluconeogenesis, ether lipid metabolism, linoleic acid metabolism, carbon fixation in photosynthetic organisms, beta-alanine metabolism, alpha-linolenic acid metabolism, quorum sensing and styrene degradation pathway were uniquely annotated in salinity stress. However, the pathways enriched only in alkalinity stress are mostly related to immunity and disease. Under the salinity-alkalinity stress, mostly pathways were enriched related to metabolism and immunity. The results suggested that compared with salinity stress, alkalinity stress makes tilapia more susceptible to pathogen infection, which more likely leads to diseases.

**Table 4:** KEGG pathway analysis of DEGs expressed only in group S, not in other groups

Pathway ID	Pathway	DEGs with pathway annotation (642)	*P-*value
ko04060	Cytokine-cytokine receptor interaction	41(6.39%)	1.13E-04
ko04512	ECM-receptor interaction	22(3.43%)	5.02E-04
ko00010	Glycolysis / Gluconeogenesis	12(1.87%)	1.29E-03
ko04978	Mineral absorption	11(1.71%)	1.65E-03
ko00565	Ether lipid metabolism	10(1.56%)	6.13E-04
ko00591	Linoleic acid metabolism	7(1.09%)	1.76E-03
ko00710	Carbon fixation in photosynthetic organisms	7(1.09%)	4.79E-05
ko00410	beta-Alanine metabolism	7(1.09%)	7.75E-04
ko00592	alpha-Linolenic acid metabolism	6(0.93%)	5.04E-04
ko02024	Quorum sensing	5(0.78%)	4.65E-04
ko00524	Neomycin, kanamycin and gentamicin biosynthesis	3(0.47%)	9.73E-04
ko00643	Styrene degradation	2(0.31%)	7.83E-04

**Table 5:** KEGG pathway analysis of DEGs expressed only in group A, not in other groups

Pathway ID	Pathway	DEGs with pathway annotation (295)	*P-*value
ko05164	Influenza A	28(9.49%)	5.35E-04
ko04145	Phagosome	22(7.46%)	6.31E-04
ko05202	Transcriptional misregulation in cancer	22(7.46%)	4.04E-04
ko05133	Pertussis	16(5.42%)	2.83E-03
ko04668	TNF signaling pathway	15(5.08%)	9.31E-05
ko04380	Osteoclast differentiation	15(5.08%)	2.81E-03
ko05145	Toxoplasmosis	14(4.75%)	2.22E-03
ko04640	Hematopoietic cell lineage	14(4.75%)	1.00E-04
ko05222	Small cell lung cancer	13(4.41%)	6.04E-04
ko05322	Systemic lupus erythematosus	12(4.07%)	1.21E-03
ko05150	*Staphylococcus aureus* infection	11(3.73%)	5.12E-04
ko04657	IL-17 signaling pathway	11(3.73%)	3.98E-04
ko05130	Pathogenic Escherichia coli infection	8(2.71%)	1.33E-03
ko05321	Inflammatory bowel disease (IBD)	8(2.71%)	2.66E-03
ko05340	Primary immunodeficiency	7(2.37%)	2.76E-05
ko01523	Antifolate resistance	6(2.03%)	4.41E-04
ko00603	Glycosphingolipid biosynthesis - globo and isoglobo series	4(1.36%)	1.23E-03

**Table 6:** KEGG pathway analysis of DEGs expressed only in group SA, not in other groups

Pathway ID	Pathway	DEGs with pathway annotation (293)	*P-*value
ko00240	Pyrimidine metabolism	10(3.41%)	8.46E-04
ko05144	Malaria	9(3.07%)	1.24E-05
ko00760	Nicotinate and nicotinamide metabolism	7(2.39%)	5.73E-04
ko05340	Primary immunodeficiency	5(1.71%)	1.27E-03
ko00500	Starch and sucrose metabolism	5(1.71%)	1.13E-03
ko00521	Streptomycin biosynthesis	4(1.37%)	5.95E-05
ko00640	Propanoate metabolism	4(1.37%)	1.29E-03
ko01210	2-Oxocarboxylic acid metabolism	4(1.37%)	4.26E-04
ko00524	Neomycin, kanamycin and gentamicin biosynthesis	3(1.02%)	4.86E-05
ko00770	Pantothenate and CoA biosynthesis	3(1.02%)	1.90E-03
ko00254	Aflatoxin biosynthesis	2(0.68%)	1.14E-03
ko00363	Bisphenol degradation	1(0.34%)	0.00E+ 00
ko02026	Biofilm formation - Escherichia coli	1(0.34%)	1.03E-03

**Table 7:** KEGG pathway analysis of DEGs expressed only in group S & A, not in other groups

Pathway ID	Pathway	DEGs with pathway annotation (238)	*P-*value
ko04970	Salivary secretion	21(8.82%)	1.12E-05
ko05416	Viral myocarditis	16(6.72%)	9.85E-05
ko04940	Type I diabetes mellitus	13(5.46%)	1.95E-04
ko05320	Autoimmune thyroid disease	13(5.46%)	2.31E-04
ko05330	Allograft rejection	13(5.46%)	1.09E-04
ko05332	Graft-versus-host disease	13(5.46%)	8.29E-05
ko04612	Antigen processing and presentation	11(4.62%)	2.46E-03
ko04640	Hematopoietic cell lineage	10(4.20%)	1.79E-03
ko05322	Systemic lupus erythematosus	10(4.20%)	1.95E-03
ko04672	Intestinal immune network for IgA production	8(3.36%)	1.71E-03
ko00590	Arachidonic acid metabolism	7(2.94%)	3.84E-04
ko03050	Proteasome	7(2.94%)	3.75E-07
ko05310	Asthma	5(2.10%)	2.10E-03
ko02026	Biofilm formation - Escherichia coli	1(0.42%)	6.84E-04
ko00643	Styrene degradation	1(0.42%)	2.34E-03

**Table 8:** KEGG pathway analysis of DEGs expressed only in group S & SA, not in other groups

Pathway ID	Pathway	DEGs with pathway annotation (97)	*P* - value
ko04971	Gastric acid secretion	11(11.34%)	7.79E-06
ko04972	Pancreatic secretion	8(8.25%)	0.001196
ko05214	Glioma	7(7.22%)	0.001021
ko04976	Bile secretion	6(6.19%)	0.000925
ko04745	Phototransduction - fly	6(6.19%)	0.001526
ko02010	ABC transporters	5(5.15%)	0.000153
ko00512	Mucin type O-glycan biosynthesis	4(4.12%)	0.000611
ko00910	Nitrogen metabolism	2(2.06%)	0.000775
ko00640	Propanoate metabolism	2(2.06%)	0.002456
ko02020	Two-component system	2(2.06%)	0.000155
ko00254	Aflatoxin biosynthesis	1(1.03%)	0.0022
ko00627	Aminobenzoate degradation	1(1.03%)	0.00193

**Table 9:** KEGG pathway analysis of DEGs expressed only in group A & SA, not in other groups

Pathway ID	Pathway	DEGs with pathway annotation (300)	P-value
ko04514	Cell adhesion molecules (CAMs)	38(12.67%)	7.04E-06
ko04144	Endocytosis	38(12.67%)	5.67E-05
ko05168	Herpes simplex infection	38(12.67%)	2.46E-10
ko05169	Epstein-Barr virus infection	37(12.33%)	8.96E-11
ko05203	Viral carcinogenesis	37(12.33%)	1.99E-09
ko04145	Phagosome	34(11.33%)	1.88E-09
ko05166	HTLV-I infection	34(11.33%)	2.38E-05
ko05416	Viral myocarditis	34(11.33%)	2.91E-14
ko04612	Antigen processing and presentation	30(10.00%)	3.52E-14
ko05320	Autoimmune thyroid disease	29(9.67%)	1.94E-13
ko04940	Type I diabetes mellitus	28(9.33%)	7.70E-13
ko05330	Allograft rejection	28(9.33%)	1.86E-13
ko05332	Graft-versus-host disease	28(9.33%)	9.75E-14
ko04650	Natural killer cell mediated cytotoxicity	27(9.00%)	1.50E-08
ko04142	Lysosome	12(4.00%)	2.26E-03
ko04110	Cell cycle	12(4.00%)	1.37E-03
ko04978	Mineral absorption	8(2.67%)	2.40E-04
ko05130	Pathogenic Escherichia coli infection	8(2.67%)	1.49E-03
ko00531	Glycosaminoglycan degradation	6(2.00%)	8.14E-06
ko00600	Sphingolipid metabolism	6(2.00%)	3.45E-03
ko00260	Glycine, serine and threonine metabolism	5(1.67%)	1.06E-03
ko03030	DNA replication	5(1.67%)	1.61E-04
ko00680	Methane metabolism	4(1.33%)	4.44E-03
ko03420	Nucleotide excision repair	4(1.33%)	4.20E-03
ko03430	Mismatch repair	4(1.33%)	3.88E-04
ko00511	Other glycan degradation	3(1.00%)	3.76E-03
ko02026	Biofilm formation - Escherichia coli	1(0.33%)	1.08E-03

**Table 10:** KEGG pathway analysis of DEGs expressed only in group S & A & SA, not in other groups

Pathway ID	Pathway	DEGs with pathway annotation (512)	P-value
ko04110	Cell cycle	30(5.86%)	4.13E-10
ko05410	Hypertrophic cardiomyopathy (HCM)	29(5.66%)	1.61E-05
ko05414	Dilated cardiomyopathy	28(5.47%)	0.000175
ko00240	Pyrimidine metabolism	27(5.27%)	2.40E-11
ko00230	Purine metabolism	26(5.08%)	0.001102
ko04111	Cell cycle - yeast	22(4.30%)	8.63E-10
ko04657	IL-17 signaling pathway	17(3.32%)	9.87E-05
ko03030	DNA replication	17(3.32%)	1.04E-15
ko00512	Mucin type O-glycan biosynthesis	16(3.12%)	1.26E-07
ko04113	Meiosis - yeast	14(2.73%)	1.49E-05
ko01524	Platinum drug resistance	13(2.54%)	0.001395
ko00480	Glutathione metabolism	13(2.54%)	0.002834
ko01200	Carbon metabolism	13(2.54%)	0.004116
ko00520	Amino sugar and nucleotide sugar metabolism	13(2.54%)	7.96E-06
ko01230	Biosynthesis of amino acids	12(2.34%)	0.000464
ko00260	Glycine, serine and threonine metabolism	11(2.15%)	5.98E-07
ko03460	Fanconi anemia pathway	9(1.76%)	0.00086
ko03430	Mismatch repair	8(1.56%)	9.24E-07
ko00680	Methane metabolism	8(1.56%)	8.77E-05
ko03440	Homologous recombination	8(1.56%)	0.000308
ko00052	Galactose metabolism	7(1.37%)	0.002908
ko03420	Nucleotide excision repair	7(1.37%)	0.000446
ko00100	Steroid biosynthesis	7(1.37%)	1.35E-06
ko03050	Proteasome	5(0.98%)	0.003347
ko00630	Glyoxylate and dicarboxylate metabolism	4(0.78%)	0.004109
ko00900	Terpenoid backbone biosynthesis	4(0.78%)	0.001549
ko00643	Styrene degradation	2(0.39%)	0.000404
ko00909	Sesquiterpenoid and triterpenoid biosynthesis	2(0.39%)	1.24E-05
ko00660	C5-Branched dibasic acid metabolism	1(0.20%)	0.000536

### The validation of differently expressional genes by qRT-PCR

**Fig. 6:**
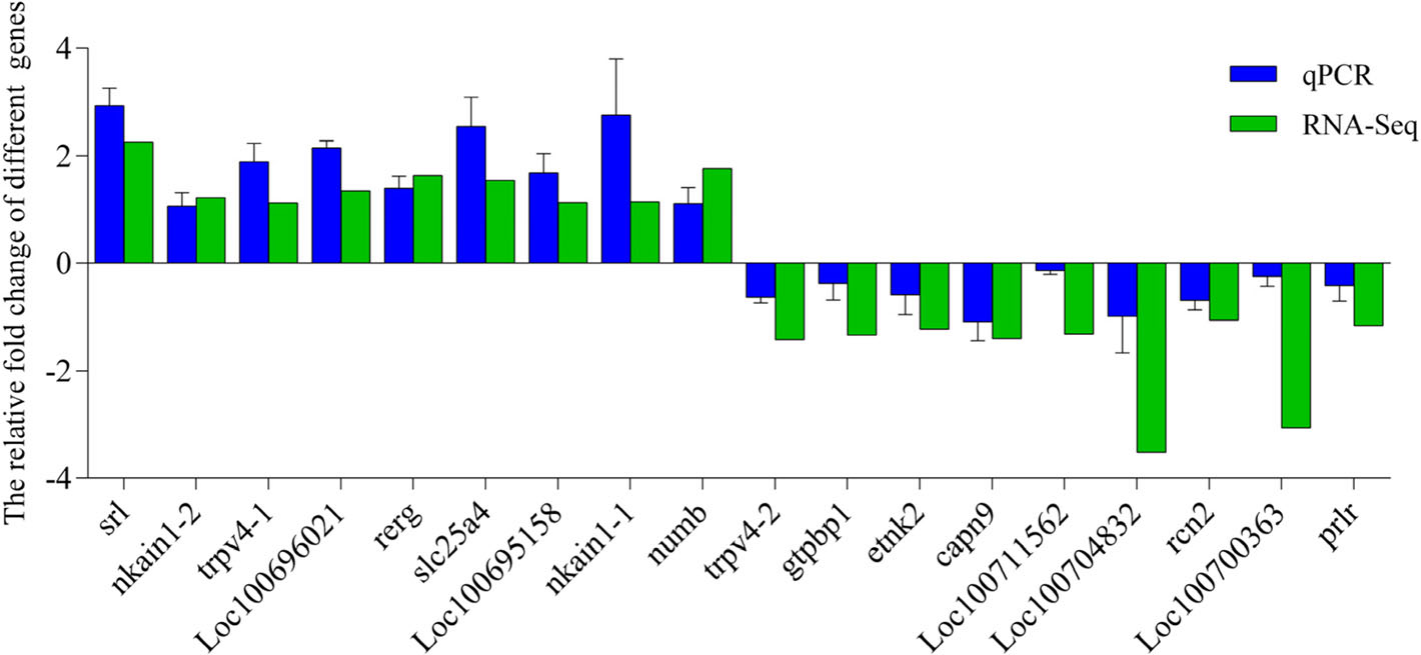
Validation of RNA-seq data by qPCR. To validate the RNA-seq data, the relative mRNA levels of 17 randomly selected DEGs in the gills of hybrid tilapia were examined by qPCR. The mRNA levels by qPCR are presented as the fold change compared with the mock-treated control after normalization against *β* -actin. The relative expression levels from the RNA-seq analysis were calculated as log_2_FC values. The gene nkai1 was calculated in two different groups. (Note: nkain1–1 was in a group C vs. A, nkain1–2 was in group C vs. SA; trpv4–1 was in group S vs. SA, trpv4–2 was in group C vs. S)

To validate the veracity and reliability of differentially expressed genes identified by mRNA-Seq, we randomly selected 17 genes for qRT-PCR validation from those with different expression patterns based on functional enrichment and pathway results. Melting-curve analysis revealed a single product for all tested genes. Log_2_FCs from qRT-PCR were compared with the mRNA-Seq expression analysis results (Fig. 6). The results of qRT-PCR were significantly correlated with the mRNA-Seq results with a correlation coefficient of 0.88 (*P* < 0.01) demonstrating the credibility of the mRNA-Seq results.

## Discussion

Osmoregulation in tilapia is a complex process because of the diverse range of salinities and alkalinities that they are exposed to in their natural habitat. To be euryhaline, tilapia must be able to cope with salt gain and osmotic water loss when it is in hyper-osmotic water stress. Edwards et al discussed the principles of ion and water transport in euryhaline fishes, and discussed the role of including gills, kidney, urinary bladder, gastrointestinal tract, rectal gland of elasmobranchs, skin, opercular membrane, and yolk sac organs in regulated the osmotic stress [38]. N’Golo et al reported the changes of ionocyte morphology and function after transfer from fresh to hypersaline waters in the tilapia *Sarotherodon melanotheron* [39].

High-throughput RNA-seq is a good method in illuminating the underlying molecular mechanisms of osmoregulation and immunization [40, 41], and the transcriptomic analysis with osmotic stress has been realized in some aquatic vertebrate species and invertebrate species, such as *Oreochromis mossambicus* [42], *Acipenser baeri* [35], *Oryzias latipes* [43], *Litopenaeus vannamei* [13, 31], *Eriocheir sinensis* [32], *Crassostrea gigas* [44] and *Mytilus trossulus* [34]. However, most of these studies were directed to only one osmotic stress treatment, but the natural water environment is complex and changing, so it is important to perform various osmotic stress treatments on fish. In this study, we conducted high-throughput sequencing of three osmotic stresses, including saline stress, alkaline stress and mixed of saline-alkaline stress on hybrid tilapia, in order to understand the osmoregulation mechanism of tilapia under different osmotic stress.

In the present study, 3772, 3412 and 3050 significantly different expression genes were identified in salinity stress, alkalinity stress and saline-alkaline stress, respectively. GO and KEGG analysis were conducted to identified genes and pathways that related to osmoregulation. GO analysis results indicated that cellular process, cell part and binding were the dominant group in the category of biological processes, cellular components and molecular functioning, respectively. These results were consistent with the researches in aquatic animals such as Pacific White Shrimp *Litopenaeus vannamei* [31], Siberian sturgeon *Acipenser baeri* [35], *Eriocheir sinensis* [32] and *Ruditapes philippinarum* [39]. Numerous ion transport enzymes and ion transporters associated with osmoregulation were identified in the three osmotic stress groups. Osmoregulation related genes, such as NKA, PRL, PRLR, TRPV4, AQP1, NHE3, and solute carrier family have been reported in many studies. Zhu et al found that the NKAα1 mRNA expression levels in the gills of tilapia reached peak level at 24 h after transfer from freshwater to seawater [45]. Yoko et al research showed that PRLR2 facilitates acclimation responses to increased extracellular osmolality [46]. Yuan et al observed the synchronous expression trend of the renal PRLR with pituitary PRL (5d) and the asynchronous expression peaks between branchial (8d) and renal PRLR (5d) in olive flounder *Paralichthys olivaceus*, and the mRNA levels of PRL receptor (PRLR) in both gill and kidney displayed a similar trend to the pituitary PRL [47]. Andre et al reported that TRPV4 expression increased by 48 h after transfer from freshwater to seawater and declined as early as 6 h after transfer from seawater to freshwater, indicating TRPV4 expression maybe regulated through the same second messenger pathways involved in hyposmotically-induced PRL release [48]. Muhammad et al suggested that cytoplasmic carbonic anhydrase (ChqCAc) gene is involved in response to changes in pH and in systemic acid–base balance in freshwater crayfish [49]. Furthermore, gene NHE3 was detected and analyzed in *Sciaenops ocellatus* [50] and *Macrobrachium* species [51]. Some other genes slc12a1, NKCC2 and AQP1 were also detected and analyzed in *Oryzias latipes* after transfer from fresh water to seawater [52]. Tatsuya et al also found Na^+^ / K^+^ − ATPase activity increased in specific gills of *Octopus ocellatus* after transfer from 30-ppt normal seawater to 20-ppt salinity [53].

By matching to KEGG pathway database, possible functions of the significant DEGs were analyzed in order to learn more about the physiological responses of hybrid tilapia to simultaneous salinity stress, alkalinity stress and salinity-alkalinity stress. 39, 51 and 58 KEGG pathways were found in salinity stress, alkalinity stress and salinity-alkalinity stress, respectively. 25 pathways were enriched in the three osmotic stress group. Among these pathways, seven pathways were related to metabolism, eight pathways were related to disease and immunity, and the remaining was related to biosynthesis, cell cycle and proximal tubule bicarbonate reclamation. In deal with hyper-osmotic stress and the energetically demanding salt extrusion, signaling related to DNA replication, mis-match repair, protein biosynthesis and degradation, metabolism and cell cycle regulation were increased [42]. Jiang et al research indicated that amino acid metabolism played crucial roles in osmoregulation [54].

In the proximal tubule bicarbonate reclamation pathway of salinity stress, the gene expression of carbonic anhydrase 4 (CA4), aquaporin-1 (AQP1), sodium-coupled neutral amino acid transporter (SNAT3, solute carrier family 38 member 3), malate dehydrogenase (MDH1) and phosphoenolpyruvate carboxykinase (GTP) (PEPCK) were significantly upregulated. However, the expression of sodium bicarbonate cotransporter (NBC1, solute carrier family 4 member 4) was significantly decreased. While in the alkalinity stress, the expression of NBC1 was significantly up-regulated, but the expression of NHE3 was similar with the control group. However, in salinity-alkalinity stress, the expression of NHE3 was significantly down-regulated, NBC1 gene was up-regulated, but the expression of AQP1 was similar to the control group. Those genes plays pivotal role in maintaining the balance of proximal tubular cell and the regulation of blood pH. The renal proximal tubule is responsible for most of the renal sodium, chloride, and bicarbonate reabsorption [55]. Alexander et al showed that NHE3 participates in the reabsorption of Na^+^, bicarbonate, and water from the proximal tubule, contributing to the maintenance of intravascular volume, blood pressure, and acid-base homeostasis [56]. Masashi et al found that a majority of renal proximal bicarbonate absorption is accomplished by coordinated operation of the apical NHE3 and the basolateral electrogenic Na^+^/ HCO_3_^−^ cotransporter NBC1 [57]. Defects in NHE3 or NBC1 can theoretically reduce the reabsorption of filtered HCO_3_^−^ and cause proximal renal tubular acidosis (pRTA) [58–61]. Gordon et al reported that AQP1 enhances the reabsorption of HCO_3_^−^ by the renal proximal tubule by increasing the CO_2_ permeability of the apical membrane and the macroscopic cotransport of Na^+^ plus two HCO_3_^−^ occurs as NBC transports Na^+^ plus CO_3_^2−^ and AQP1 transports CO_2_ and H_2_O [62]. Our results indicated that proximal tubule bicarbonate reclamation pathway was evidently crucial for maintaining systemic acid-base homeostasis in osmotic stress of tilapia.

Nine pathways were uniquely enriched in salinity stress, such as ECM-receptor interaction, protein processing in endoplasmic reticulum, nitrogen metabolism and arachidonic acid metabolism. Among these pathways, arachidonic acid metabolism playing important role in osmoregulation has been reported in *Eriocheir sinensis* [32], *Litopenaeus vannamei* [31], *Ruditapes philippinarum* [39] and *Litopenaeus vannamei* [63]. Six KEGG pathways were characteristic enriched in alkalinity stress group. Meanwhile, 12 KEGG pathways, including pantothenate and CoA biosynthesis, mineral absorption, valine, leucine and isoleucine biosynthesis, cell adhesion molecules (CAMs), glyoxylate, dicarboxylate metabolism and propanoate metabolism, etc., were uniquely annotated in salinity-alkalinity stress. In the mineral absorption pathway, TRPV6, chloride channel 2 (clcn2), zinc transporter (ZnT1), Na^+^ / K^+^-exchanging ATPase and femitin (FTH1) were upregulated significantly. Meanwhile, NHE3 and heme oxygenase 1 (HMOX1) were downregulated significantly. Through the network formed of all genes, this pathway played vital role in ion regulation and maintain the balance of osmotic stress in internal and external of the tilapia.

From KEGG pathway analysis of significant DEGs uniquely in salinity stress group, three enriched pathways related to lipid metabolism including alpha-linolenic acid metabolism, linoleic acid metabolism and ether lipid metabolism were examined. The long-chain polyunsaturated fatty acid (LC-PUFA) level raised in euryhaline and migratory teleosts during salinity acclimation has been reported by many researches, such as *Oncorhynchus mykiss* [64], *Dicentrarchus labrax* [65], and *Oncorhynchus masou* [66]. LC-PUFAs, as structural components of plasma membrane, enhanced the fluidity of cell membranes [67], results in the changes of other physical membrane properties and affects the membrane-protein binding [68]. These results could be used to explain the increase of LC-PUFAs level in euryhaline and migratory teleosts during salinity acclimation. While in alkalinity stress, the enriched pathways were mostly related to disease, pathogen defense and innate immune response, suggesting that tilapia was more vulnerable to alkaline stress than saline stress.

While in salinity-alkalinity stress group, most pathways were related to biosynthesis and metabolism, such as pantothenate and CoA biosynthesis, neomycin, kanamycin and gentamicin biosynthesis, nicotinate and nicotinamide metabolism and 2-Oxocarboxylic acid metabolism. In saline & alkaline, the enriched pathways of significant DEGs mostly related to antigen processing and antibody production. In saline & saline-alkaline, most pathways were related to secretion and metabolism, such as gastric acid secretion, pancreatic secretion, nitrogen metabolism and propanoate metabolism. While in alkaline & saline-alkaline, most pathways were related to immunity, and mismatch repair, nucleotide excision repair, DNA replication, glycine, serine and threonine metabolism, cell cycle, sphingolipid metabolism, glycosaminoglycan degradation, and so on, which were related to osmoregulation. Inokuchi et al showed that the apoptosis of freshwater-type mitochondrial rich cells would be enhanced and stimulated the recruitment of mitochondrial rich-cell complexes to develop hypo-osmoregulatory ability for seawater adaptation after transfer from hypo-osmotic to hyper-osmotic water in *Oreochromis mossambicus* [69]. Moreover, osmotic stress has been reported to cause oxidative stress, protein damage, DNA double-strand breaks and oxidative base modification [70–72]. These results are in consistent with the enrichment of canonical pathways such as mismatch repair, nucleotide excision repair, DNA replication, glycine, serine and threonine metabolism and cell cycle in response to osmotic tolerance of the hybrid tilapia. In saline & alkaline & salinity-alkalinity, 28 pathways were found, of which 10 pathways were related to metabolism, such as glutathione metabolism, glycine, serine and threonine metabolism, purine metabolism and pyrimidine metabolism.

## Conclusions

Here, the transcriptome of the hybrid tilapia response to saline stress, alkaline stress and saline-alkaline stress was examined following the application of RNA-seq. Through more than 40 million sequence reads and the expression data of the four libraries, the study provided some useful insights into signal transduction pathways in hybrid tilapia and offered a number of candidate genes as potential markers of tolerance to osmotic stress for tilapia. The present research described for the first time the effects of salinity, alkalinity and salinity-alkalinity on gilled classic osmoregulation-related genes and signaling pathways during acclimation to saline and alkaline environment in tilapia. Our results provide a deep insight into the gill osmotic stress response of the hybrid tilapia to saline stress, alkaline stress and the mix of saline-alkaline stress that will be helpful in identifying reliable osmoregulation target genes and pathways for fish in osmotic stress.

## Methods

### Sample collection

The gill tissue samples were obtained from the hybrid tilapia (*O. mossambicus♀* × *O. urolepis hornorum♂*) specimens at the Pearl River Fisheries Research Institute, Chinese Academy of Fishery Sciences, Guangzhou, China. All fishes were acclimated in the laboratory environment (28 °C) for two weeks before the experiment. The average weight of the experimental fishes was 51.27 ± 3.78 g. Three treatments with three replicates each were set up. The tilapias were divided into three groups (90 tilapias per group) and acclimated in saline stress (S, 25 ‰), alkali stress (A, 4 ‰) and saline-alkali stress (SA, S: 15 ‰, A: 4 ‰) at 28 °C, respectively. The control group was maintained in fresh water (saline, 0 ‰; alkali, 0 ‰). After 24 h (h) of treatment, 18 tilapias from each group were randomly selected and anesthetized by immersion in 100 mg/L MS-222 (Sigma, MO, USA) diluted in aquarium water. Following anesthesia, the fishes were decapitated and the gills were dissected immediately and immersed into liquid nitrogen for high-throughput sequencing. After 24 h of osmotic stress, the remaining fishes were released.

### RNA isolation, library construction and illumina sequencing

The TRIzol® reagent (Invitrogen, USA) was used to extract the total RNA from four samples according to the manufacturer’s protocol and then treated by DNase I to implement the DNA digestion and obtained pure RNA products. Finally, the integrity of the total RNA was confirmed using 1% agarose gel electrophoresis and an Agilent 2100 Bio analyzer (Agilent Technologies, USA). Equal amounts of RNA from different individuals in the same group were pooled for library construction. Next generation sequencing library preparations were constructed according to the manufacturer’s protocol (NEBNext® Ultra™ RNA Library Prep Kit for Illumina®). Subsequently, the deep sequencing of the four mRNA libraries was performed using Illumina HiSeq 2000 according to the manufacturer’s instructions at the GENEWIZ (Genewiz, Suzhou, China).

### Basic analysis of sequencing data

In order to remove technical sequences, including adapters, polymerase chain reaction (PCR) primers, or fragments thereof, and the quality of bases lower than 20, the pass filter data in the fastq format were processed by Trimmomatic (v0.30) to provide high-quality, clean data. Firstly, the reference genome sequences and the gene model annotation files of relative species were downloaded from genome website, such as NCBI. Secondly, Hisat2 (v2.0.1) was used to indexed with reference genome sequence. Finally, we mapped the clean reads to reference sequences of *Oreochromis niloticus* (National Center for Biotechnology Information, NCBI; accession numbers: GCA_001858045.3, https://www.ncbi.nlm.nih.gov/assembly/GCA_001858045.3) via software Hisat2 (v2.0.1). In the beginning transcripts in fasta format are converted from known gff annotation file and indexed properly. Then, with the file as a reference gene file, HTSeq (v0.6.1) estimated gene and isoform expression levels from the pair-end clean data.

### Differential expression analysis and bioinformatics analysis of genes

DESeq2 and edegR method were used to conducted differential expression analysis. FPKM (Fragments Per Kilobases per Millionreads) method can eliminate the influence of gene length and sequencing amount on the calculated gene expression, and the calculated gene expression level can be directly used to compare the expression differences between different genes, so it was used to normalized the data. After adjusting by BH (fdr correction with Benjamini/Hochberg) for controlling the false discovery rate, the *P*-values < 0.05 and |log2FC| > = 1 were set to detect significant differentially expressed ones [73, 74]. The gene ontology (GO) enrichment analysis indicated that the significant differentially expressed genes with a significant *P-value* less than 0.05 were enriched for biological processes, molecular functioning, and cellular components through the software GO-TermFinder. KEGG (Kyoto Encyclopedia of Genes and Genomes) is a collection of databases dealing with genomes, biological pathways, diseases, drugs, and chemical substances (https://www.genome.jp/kegg/kegg3.html) [75]. We used scripts in house to enrich significant differential expression gene in KEGG pathways. The Venny online software (http://bioinfogp.cnb.csic.es/tools/venny/) was used to combine the analyses of differentially expressed genes. Gene Set Enrichment Analysis (GSEA) was also used to detect the expression changes of the whole gene set. This enrichment tool can fully detect genes that are not significantly different in expression levels but are plays important biological role at the overall level.

### RNA-Seq data validation by qPCR and statistical analysis

To validate and measure the differential expression of mRNAs identified by high-throughput sequencing, 16 differentially expressed mRNAs were randomly selected for real-time quantitative PCR analyses. Real-time quantitative PCR was performed using the SYBR Green PCR Master Mix (Life Technologies, USA) according to the manufacture’s protocol in an ABI 7300 Real-Time PCR System (Applied Biosystems, Foster City, CA) using the following conditions: 50 °C for 2 min, 95 °C for 2 min, 40 cycles of 95 °C for 15 s, 60 °C for 15 s and 72 °C 30s. At the end of qPCR assay, the melting curve was analyzed to evaluate the amplification specificity. All primer sequences are listed in Table 11. The relative expression was calculated using the 2^-△△Ct^ method and *β*-actin was used as the internal control to normalize mRNAs expression. The results are presented as the mean ± standard error. The analysis of all data was implemented using the statistical software SPSS 22.0 (SPSS, Chicago, IL, USA) and the expression levels of mRNAs were analyzed using one-way ANOVA method.

**Table 11:** Primers of different expression genes used for the qRT-PCR analysis

Genes	Forward primer sequence(5′-3′)	Reverse primer sequence(5′-3′)
*srl*	TTCCTGGGACCGTGGAGTGT	GTCAGCCGCCATAACGATAC
*gtpbp1*	TGTGGCAGCATAAGGCAGAC	GGTTCCTACAGCCTTGGTGC
*etnk2*	TAACGGAAGGAGCGATGAAG	ATACTCGGACCAGGACTACG
*capn9*	AGACCATTGGCTTTGCTGTG	GTGGGAACCAGCAGGTAGTG
*rerg*	CAGGAGGACATCCAGCAGAG	CCAGGTCAGACTTGTTGCCC
*slc25a4*	GCAGTCTGGACGCAAAGGAG	TCATCGTAAAGCACCAGCAC
LOC100695158	TCTACAGGAGCAAAGGGAGG	CTTGGTTCTTGTCATCGGAG
LOC100711562	GTGTCTTCACGCCTCTTCCT	CTCGTCCCGTTTGTAGGCAT
*numb*	CATCTCCCTTCAAACGACAG	CACTGAAGGCTGTGGCGATT
LOC1007004832	GTGGTGCTAAACAAAGTCCG	ATGCCCACGACATCCTCTTC
*rcn2*	GAATACAACACGGTGGCTCA	GCCATCCACATCAGCAAAGT
LOC100700363	TGGTGGACAGACAGAAGTGC	CTCCATCACACAACAGCGGC
*nkai1*	TTCAACACATCCCTCCATCG	CCAAAGAGAGCCAAGAGGAT
*prlr*	GCACTGGGCAAGACCTACTC	TACGAGGTGGTTCCCAAGAC
*trpv4*	CCAGTCACAATGAGAACCGC	GTATGTGGGTATGGAGGCTT
LOC100696021	TCTGCTAAACCTGCTCACCG	ATAGTTCAGCCCACAGACCC

## Supplementary information

**Additional file 1.** Summary results of significant DEGs analysis and the results of GO, KEGG enrichment.

## Data Availability

All the data supporting our findings are contained within the manuscript. The clean sequence reads dataset supporting the conclusions of this article are available on NCBI BioProject with accession number PRJNA558948 (https://www.ncbi.nlm.nih.gov/bioproject/PRJNA558948) and will be made publicly available upon acceptance of this manuscript for publishing.

## Abbreviations

A: Alkaline stress group
bp: Base pairs (measuring unit)
C: Control group
DEGs: Differentially expressed genes
FPKM: Fragments Per Kilobases per Millionreads
GO: Gene ontology
GSEA: Gene Set Enrichment Analysis
KEGG: Kyoto encyclopedia of genes and genomes
KO: KEGG ortholog database
qRT-PCR: Quantitative Real Time Polymerase Chain Reaction
RNA-Seq: RNA-sequencing
S: Saline stress group
SA: Saline-Alkaline group
