# Supplementary material for: Transcriptomic response to three osmotic stresses in gills of hybrid tilapia (Oreochromis mossambicus female × O. urolepis hornorum male)
Source: BMC Genomics. 2020 Jan 31;21:110. doi: 10.1186/s12864-020-6512-5 (PMC6995152; doi:10.1186/s12864-020-6512-5)
Supplement: Supplementary file 1 — Additional file 1. Summary results of significant DEGs analysis and the results of GO, KEGG enrichment. [file 12864_2020_6512_MOESM1_ESM.docx]

**Fig. S1** Volcano plot of differentially expressed genes (DEGs) in tilapia gills by comparing different libraries. The expression levels for each DEGs was included in the volcano plot. The X-axis exhibits differences in fold changes between two sets of samples (**A**: C vs. S; **B**: C vs. A; **C**: C vs. SA; **D**: S vs. SA; **E**: A vs. SA), and the Y-axis shows the significance of the mRNAs. Red (up-regulation) and green (down-regulation) dots represent significantly different expression (*P-*value < 0.05, |log2 (fold change)| > 1), respectively, and black dots represent no significant differences.


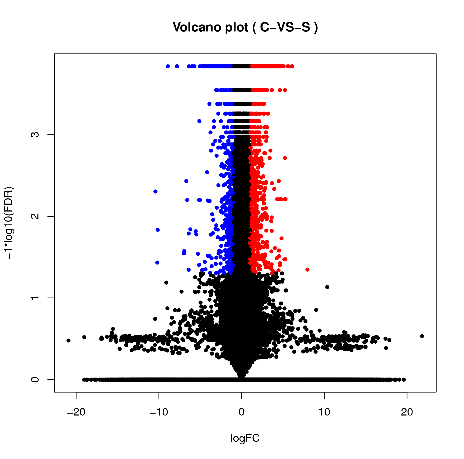

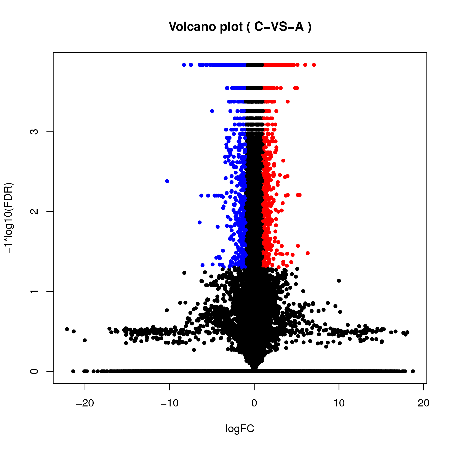

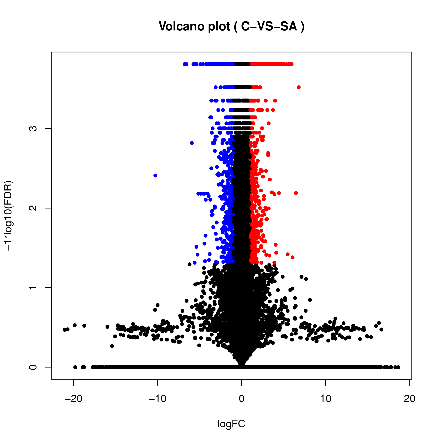

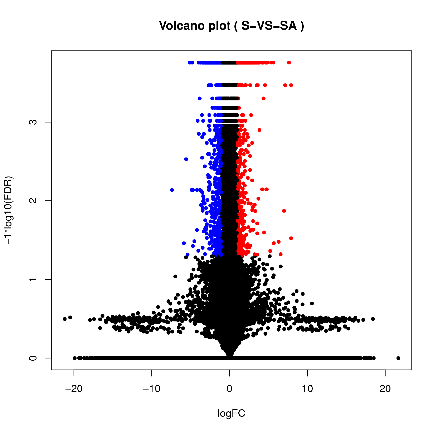

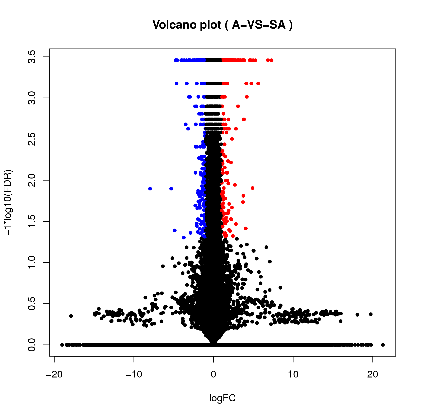


**A**

**B**

**C**

**D**

**E**

**Fig. S2** Hierarchical cluster analysis of differentially expressed genes each interval in Venn diagram. Heat map showing differentially-expressed mRNAs upon osmotic stress in hybrid tilapia. Expression heat map was generated with the value of log_10_^FPKM^. The color scale shows the levels of differentially expressed genes: red color indicates enhanced expression of mRNA and blue color indicates decrease in expression levels of mRNA. **A**: S; **B**: A; **C**: SA; **D**: S & A; **E**: S & SA; **F**: A & SA;**G**: S, A & SA.


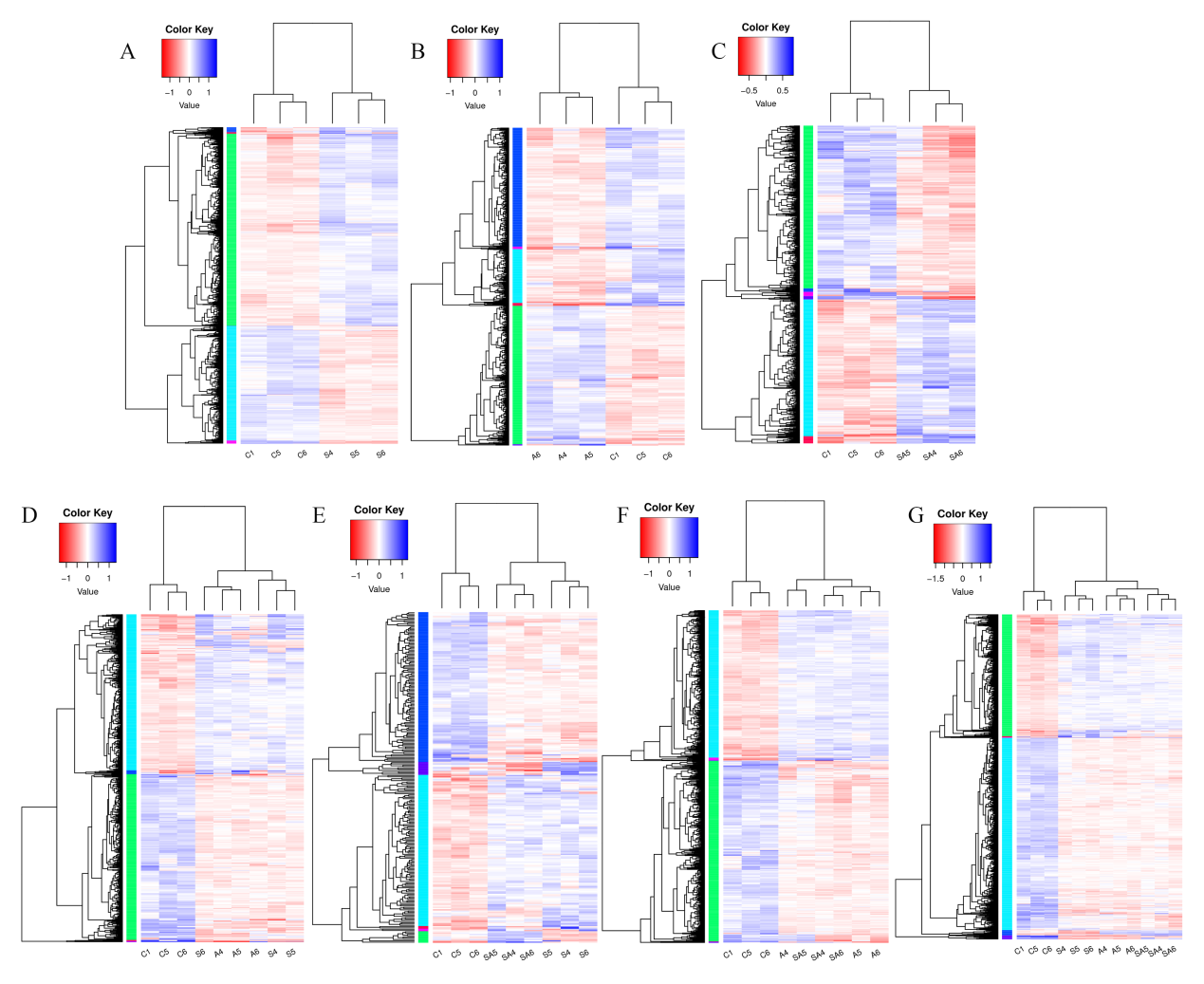


**Table S1** The top 30 KEGG pathways with the most number of annotated sequences in a group C vs. S.

| KEGG ID | Pathway description | DEGs with pathway annotation | *P-*value |
| --- | --- | --- | --- |
| ko04060 | Cytokine-cytokine receptor interaction | 71 (4.77%) | 4.66E-03 |
| ko05133 | Pertussis | 58 (3.90%) | 1.91E-03 |
| ko04141 | Protein processing in endoplasmic reticulum | 51 (3.43%) | 4.72E-04 |
| ko05320 | Autoimmune thyroid disease | 46 (3.09%) | 6.09E-04 |
| ko04940 | Type I diabetes mellitus | 46 (3.09%) | 4.24E-04 |
| ko04110 | Cell cycle | 44 (2.96%) | 4.70E-05 |
| ko04612 | Antigen processing and presentation | 44 (2.96%) | 2.07E-03 |
| ko05330 | Allograft rejection | 43 (2.89%) | 7.74E-04 |
| ko05322 | Systemic lupus erythematosus | 42 (2.82%) | 2.04E-04 |
| ko05332 | Graft-versus-host disease | 41 (2.75%) | 1.51E-03 |
| ko04512 | ECM-receptor interaction | 40 (2.69%) | 1.03E-03 |
| ko00240 | Pyrimidine metabolism | 37 (2.48%) | 7.82E-06 |
| ko01200 | Carbon metabolism | 33 (2.22%) | 2.86E-04 |
| ko04657 | IL-17 signaling pathway | 31 (2.08%) | 2.30E-03 |
| ko04111 | Cell cycle - yeast | 30 (2.01%) | 3.47E-05 |
| ko00512 | Mucin type O-glycan biosynthesis | 28 (1.88%) | 4.70E-07 |
| ko00520 | Amino sugar and nucleotide sugar metabolism | 26 (1.75%) | 1.46E-06 |
| ko01230 | Biosynthesis of amino acids | 25 (1.68%) | 3.81E-04 |
| ko04115 | p53 signaling pathway | 23 (1.54%) | 3.35E-03 |
| ko00010 | Glycolysis / Gluconeogenesis | 23 (1.54%) | 3.89E-04 |
| ko00051 | Fructose and mannose metabolism | 22 (1.48%) | 0.00013 |
| ko03030 | DNA replication | 20 (1.34%) | 1.37E-10 |
| ko00590 | Arachidonic acid metabolism | 20 (1.34%) | 0.00297 |
| ko05310 | Asthma | 16 (1.07%) | 0.00338 |
| ko04964 | Proximal tubule bicarbonate reclamation | 16 (1.07%) | 0.000791 |
| ko00260 | Glycine, serine and threonine metabolism | 16 (1.07%) | 4.43E-05 |
| ko03050 | Proteasome | 15 (1.01%) | 2.97E-06 |
| ko00250 | Alanine, aspartate and glutamate metabolism | 15 (1.01%) | 0.00259 |
| ko00052 | Galactose metabolism | 15 (1.01%) | 0.00158 |
| ko00680 | Methane metabolism | 13 (0.87%) | 0.000745 |

**Table S2** The top 30 KEGG pathways with the most number of annotated sequences in a group C vs. A.

| KEGG ID | Pathway description | DEGs with pathway annotation | *P-*value |
| --- | --- | --- | --- |
| ko05166 | HTLV-I infection | 104 (7.73%) | 1.77E-04 |
| ko05164 | Influenza A | 95 (7.06%) | 4.30E-04 |
| ko04145 | Phagosome | 92 (6.84%) | 3.11E-09 |
| ko05203 | Viral carcinogenesis | 84 (6.25%) | 1.63E-04 |
| ko05168 | Herpes simplex infection | 83 (6.17%) | 1.00E-04 |
| ko05416 | Viral myocarditis | 77 (5.72%) | 1.80E-12 |
| ko05169 | Epstein-Barr virus infection | 74 (5.50%) | 6.03E-04 |
| ko04612 | Antigen processing and presentation | 71 (5.28%) | 3.09E-15 |
| ko05320 | Autoimmune thyroid disease | 63 (4.68%) | 1.92E-11 |
| ko04940 | Type I diabetes mellitus | 61 (4.54%) | 7.56E-11 |
| ko05330 | Allograft rejection | 60 (4.46%) | 1.99E-11 |
| ko05332 | Graft-versus-host disease | 60 (4.46%) | 6.63E-12 |
| ko05133 | Pertussis | 58 (4.31%) | 1.57E-04 |
| ko00230 | Purine metabolism | 56 (4.16%) | 8.75E-04 |
| ko04650 | Natural killer cell mediated cytotoxicity | 56 (4.16%) | 5.65E-04 |
| ko04110 | Cell cycle | 49 (3.64%) | 4.22E-08 |
| ko05322 | Systemic lupus erythematosus | 43 (3.20%) | 9.17E-06 |
| ko00240 | Pyrimidine metabolism | 41 (3.05%) | 1.03E-08 |
| ko04668 | TNF signaling pathway | 39 (2.90%) | 1.20E-03 |
| ko04657 | IL-17 signaling pathway | 38 (2.83%) | 1.08E-06 |
| ko04111 | Cell cycle - yeast | 31 (2.30%) | 1.67E-06 |
| ko01524 | Platinum drug resistance | 28 (2.08%) | 0.000292 |
| ko00480 | Glutathione metabolism | 27 (2.01%) | 0.002089 |
| ko01200 | Carbon metabolism | 26 (1.93%) | 0.007155 |
| ko05130 | Pathogenic Escherichia coli infection | 25 (1.86%) | 0.000153 |
| ko00520 | Amino sugar and nucleotide sugar metabolism | 25 (1.86%) | 7.21E-07 |
| ko03030 | DNA replication | 24 (1.78%) | 2.17E-15 |
| ko04115 | p53 signaling pathway | 24 (1.78%) | 0.000374 |
| ko01230 | Biosynthesis of amino acids | 21 (1.56%) | 0.002499 |
| ko00260 | Glycine, serine and threonine metabolism | 21 (1.56%) | 4.77E-09 |

**Table S3** The top 30 KEGG pathways with the most number of annotated sequences in a group C vs. SA.

| KEGG ID | Pathway description | DEGs with pathway annotation | *P-*value |
| --- | --- | --- | --- |
| ko04514 | Cell adhesion molecules (CAMs) | 93 (7.74%) | 8.01E-03 |
| ko05166 | HTLV-I infection | 87 (7.24%) | 3.82E-03 |
| ko05203 | Viral carcinogenesis | 77 (6.41%) | 1.34E-04 |
| ko04145 | Phagosome | 73 (6.07%) | 1.04E-05 |
| ko05168 | Herpes simplex infection | 68 (5.66%) | 3.62E-03 |
| ko05169 | Epstein-Barr virus infection | 66 (5.49%) | 1.18E-03 |
| ko04612 | Antigen processing and presentation | 57 (4.74%) | 1.35E-10 |
| ko05416 | Viral myocarditis | 57 (4.74%) | 9.50E-07 |
| ko00230 | Purine metabolism | 55 (4.58%) | 9.40E-05 |
| ko05320 | Autoimmune thyroid disease | 51 (4.24%) | 4.38E-08 |
| ko04940 | Type I diabetes mellitus | 48 (3.99%) | 3.95E-07 |
| ko05133 | Pertussis | 47 (3.91%) | 4.28E-03 |
| ko05332 | Graft-versus-host disease | 47 (3.91%) | 8.17E-08 |
| ko05330 | Allograft rejection | 47 (3.91%) | 1.78E-07 |
| ko04650 | Natural killer cell mediated cytotoxicity | 46 (3.83%) | 7.70E-03 |
| ko04110 | Cell cycle | 46 (3.83%) | 2.49E-08 |
| ko05410 | Hypertrophic cardiomyopathy (HCM) | 45 (3.74%) | 2.79E-03 |
| ko00240 | Pyrimidine metabolism | 44 (3.66%) | 8.09E-12 |
| ko05322 | Systemic lupus erythematosus | 35 (2.91%) | 3.51E-04 |
| ko01200 | Carbon metabolism | 30 (2.50%) | 6.36E-05 |
| ko04657 | IL-17 signaling pathway | 29 (2.41%) | 0.000302 |
| ko04111 | Cell cycle - yeast | 28 (2.33%) | 4.15E-06 |
| ko03030 | DNA replication | 26 (2.16%) | 8.54E-19 |
| ko01230 | Biosynthesis of amino acids | 25 (2.08%) | 1.15E-05 |
| ko01524 | Platinum drug resistance | 23 (1.91%) | 0.002739 |
| ko00480 | Glutathione metabolism | 23 (1.91%) | 0.00715 |
| ko05130 | Pathogenic Escherichia coli infection | 23 (1.91%) | 0.000173 |
| ko00512 | Mucin type O-glycan biosynthesis | 22 (1.83%) | 1.13E-05 |
| ko00520 | Amino sugar and nucleotide sugar metabolism | 21 (1.75%) | 1.36E-05 |
| ko00260 | Glycine, serine and threonine metabolism | 20 (1.66%) | 3.64E-09 |

**Table S4** The results of GO terms after GSEA analysis in a group C vs. S.

ES: enrichment score, NES: normalized enrichment score, *P*-value: nominal P value, FDR: false discovery rate.

| **GO Term** | **gene set name** | **Size** | **ES** | **NES** | ***P*-value** | ***P* adjust** | **Leading edge** |
| --- | --- | --- | --- | --- | --- | --- | --- |
| Biological process | GO:1901575 | 56 | -0.30425 | -1.35733 | 0 | 0.63875 | 17 |
|  | GO:0017144 | 29 | -0.37594 | -1.28831 | 0.088176 | 0.644383 | 15 |
|  | GO:0009161 | 15 | -0.53227 | -1.36901 | 0 | 0.647742 | 8 |
|  | GO:0072330 | 15 | -0.40536 | -1.29045 | 0.091085 | 0.673772 | 6 |
|  | GO:0009156 | 15 | -0.53227 | -1.36901 | 0 | 0.697568 | 8 |
|  | GO:0006164 | 19 | -0.40923 | -1.29563 | 0 | 0.700776 | 8 |
|  | GO:0009259 | 27 | -0.40398 | -1.37457 | 0 | 0.738198 | 11 |
|  | GO:0072522 | 19 | -0.40923 | -1.29563 | 0 | 0.741999 | 8 |
|  | GO:0009152 | 19 | -0.40923 | -1.29563 | 0 | 0.788373 | 8 |
|  | GO:0019693 | 27 | -0.40398 | -1.37457 | 0 | 0.805307 | 11 |
|  | GO:0009124 | 16 | -0.50405 | -1.37529 | 0 | 0.875038 | 8 |
|  | GO:0009123 | 16 | -0.50405 | -1.37529 | 0 | 0.972264 | 8 |
|  | GO:0010564 | 17 | 0.68712 | 1.206156 | 0 | 1 | 16 |
|  | GO:1902531 | 27 | 0.328062 | 1.204494 | 0.095057 | 1 | 13 |
|  | GO:0051716 | 44 | 0.430065 | 1.191079 | 0.111546 | 1 | 31 |
|  | GO:1901605 | 21 | 0.335738 | 1.178763 | 0.218367 | 1 | 9 |
|  | GO:0051726 | 33 | 0.560228 | 1.145522 | 0.084149 | 1 | 28 |
|  | GO:0007017 | 37 | 0.472441 | 1.141973 | 0.209393 | 1 | 26 |
|  | GO:1903047 | 27 | 0.681263 | 1.131134 | 0 | 1 | 26 |
|  | GO:0033554 | 33 | 0.469234 | 1.120802 | 0.111546 | 1 | 24 |
|  | GO:0022402 | 39 | 0.606701 | 1.107205 | 0 | 1 | 35 |
|  | GO:0006508 | 20 | 0.555567 | 1.10586 | 0.287671 | 1 | 17 |
|  | GO:0042221 | 26 | 0.289603 | 1.08453 | 0.28719 | 1 | 10 |
|  | GO:0006974 | 26 | 0.511149 | 1.077573 | 0.097847 | 1 | 20 |
|  | GO:0051603 | 17 | 0.579305 | 1.075567 | 0.181996 | 1 | 15 |
|  | GO:0006520 | 30 | 0.39322 | 1.074909 | 0.203523 | 1 | 15 |
|  | GO:0006281 | 23 | 0.49052 | 1.057216 | 0.195695 | 1 | 18 |
|  | GO:0051347 | 40 | 0.498208 | 1.054749 | 0.28626 | 1 | 38 |
|  | GO:0051641 | 37 | 0.462766 | 1.054028 | 0.315068 | 1 | 26 |
|  | GO:0044265 | 15 | 0.562712 | 1.048184 | 0.181996 | 1 | 13 |
|  | GO:0031099 | 19 | 0.266347 | 1.042345 | 0.298 | 1 | 9 |
|  | GO:0051276 | 29 | 0.535416 | 1.03829 | 0.170254 | 1 | 22 |
|  | GO:0046434 | 15 | -0.49107 | -1.56953 | 0 | 1 | 8 |
|  | GO:0032787 | 21 | -0.39318 | -1.46177 | 0 | 1 | 8 |
|  | GO:0046700 | 15 | -0.38687 | -1.43487 | 0 | 1 | 6 |
|  | GO:0009150 | 25 | -0.43645 | -1.42611 | 0 | 1 | 11 |
|  | GO:0072521 | 25 | -0.43645 | -1.42611 | 0 | 1 | 11 |
|  | GO:0006163 | 25 | -0.43645 | -1.42611 | 0 | 1 | 11 |
|  | GO:1901361 | 19 | -0.42063 | -1.40543 | 0.119772 | 1 | 8 |
|  | GO:0016310 | 15 | -0.40628 | -1.39216 | 0 | 1 | 6 |
| Cellular Components | GO:0051276 | 26 | -0.68499 | -1.38123 | 0 | 1 | 21 |
|  | GO:1903047 | 27 | -0.77129 | -1.35713 | 0 | 1 | 24 |
|  | GO:0022402 | 37 | -0.66383 | -1.32777 | 0 | 1 | 28 |
|  | GO:0045786 | 15 | -0.57228 | -1.22504 | 0 | 1 | 10 |
|  | GO:0007346 | 16 | -0.62076 | -1.19657 | 0 | 1 | 11 |
|  | GO:0006260 | 24 | -0.68331 | -1.18986 | 0 | 1 | 24 |
|  | GO:0010564 | 18 | -0.60547 | -1.17065 | 0 | 1 | 16 |
|  | GO:0051716 | 46 | -0.37171 | -1.14788 | 0.383562 | 1 | 23 |
|  | GO:0006974 | 31 | -0.53278 | -1.11029 | 0.097847 | 1 | 20 |
|  | GO:0034645 | 45 | -0.403 | -1.10203 | 0.387476 | 1 | 29 |
|  | GO:0051726 | 34 | -0.50415 | -1.09879 | 0.195695 | 1 | 20 |
|  | GO:0006281 | 27 | -0.51723 | -1.09865 | 0.197652 | 1 | 17 |
|  | GO:0009059 | 50 | -0.39338 | -1.08477 | 0.485323 | 1 | 31 |
|  | GO:0033554 | 37 | -0.40792 | -1.0505 | 0.385069 | 1 | 20 |
|  | GO:0007010 | 28 | -0.21015 | -1.0416 | 0.472 | 1 | 13 |
|  | GO:0007017 | 36 | -0.41998 | -1.03523 | 0.303327 | 1 | 23 |
|  | GO:0007049 | 15 | -0.60299 | -1.02908 | 0.199609 | 1 | 11 |
|  | GO:0006310 | 17 | -0.42479 | -1.0258 | 0.390963 | 1 | 8 |
|  | GO:0051641 | 29 | -0.31716 | -1.02572 | 0.290258 | 1 | 13 |
|  | GO:0016053 | 18 | -0.26659 | -1.01839 | 0.505051 | 1 | 7 |
|  | GO:0003013 | 16 | 0.393673 | 1.518086 | 0 | 1 | 7 |
|  | GO:1901605 | 16 | 0.463673 | 1.399441 | 0 | 1 | 9 |
|  | GO:0006520 | 20 | 0.420028 | 1.341835 | 0 | 1 | 10 |
|  | GO:0009150 | 21 | 0.407713 | 1.168968 | 0.323944 | 1 | 10 |
|  | GO:1901565 | 17 | 0.352682 | 1.147959 | 0.189723 | 1 | 7 |
|  | GO:0097435 | 16 | 0.52952 | 1.135176 | 0.301498 | 1 | 12 |
|  | GO:0090066 | 16 | 0.459648 | 1.134224 | 0.191011 | 1 | 9 |
|  | GO:0010648 | 18 | 0.406477 | 1.128787 | 0.225743 | 1 | 10 |
|  | GO:0023057 | 18 | 0.406477 | 1.128787 | 0.225743 | 1 | 10 |
|  | GO:0006732 | 19 | 0.35308 | 1.12107 | 0.321792 | 1 | 11 |
|  | GO:0072521 | 22 | 0.378844 | 1.119955 | 0.484909 | 1 | 10 |
|  | GO:0006163 | 22 | 0.378844 | 1.119955 | 0.484909 | 1 | 10 |
|  | GO:0017144 | 19 | 0.536873 | 1.110253 | 0.121331 | 1 | 16 |
|  | GO:0055080 | 15 | 0.532057 | 1.092722 | 0.187867 | 1 | 10 |
|  | GO:0009968 | 16 | 0.400672 | 1.085885 | 0.315686 | 1 | 9 |
|  | GO:0048878 | 20 | 0.457171 | 1.058021 | 0.315068 | 1 | 15 |
|  | GO:0098771 | 16 | 0.521577 | 1.05715 | 0.379648 | 1 | 13 |
|  | GO:0050801 | 16 | 0.521577 | 1.05715 | 0.379648 | 1 | 13 |
|  | GO:0019693 | 23 | 0.344604 | 1.049205 | 0.423387 | 1 | 10 |
|  | GO:0009259 | 23 | 0.344604 | 1.049205 | 0.423387 | 1 | 10 |
| Molecular Function | GO:1901575 | 56 | -0.30425 | -1.35733 | 0 | 0.63875 | 17 |
|  | GO:0017144 | 29 | -0.37594 | -1.28831 | 0.088176 | 0.644383 | 15 |
|  | GO:0009161 | 15 | -0.53227 | -1.36901 | 0 | 0.647742 | 8 |
|  | GO:0072330 | 15 | -0.40536 | -1.29045 | 0.091085 | 0.673772 | 6 |
|  | GO:0009156 | 15 | -0.53227 | -1.36901 | 0 | 0.697568 | 8 |
|  | GO:0006164 | 19 | -0.40923 | -1.29563 | 0 | 0.700776 | 8 |
|  | GO:0009259 | 27 | -0.40398 | -1.37457 | 0 | 0.738198 | 11 |
|  | GO:0072522 | 19 | -0.40923 | -1.29563 | 0 | 0.741999 | 8 |
|  | GO:0009152 | 19 | -0.40923 | -1.29563 | 0 | 0.788373 | 8 |
|  | GO:0019693 | 27 | -0.40398 | -1.37457 | 0 | 0.805307 | 11 |
|  | GO:0009124 | 16 | -0.50405 | -1.37529 | 0 | 0.875038 | 8 |
|  | GO:0009123 | 16 | -0.50405 | -1.37529 | 0 | 0.972264 | 8 |
|  | GO:0010564 | 17 | 0.68712 | 1.206156 | 0 | 1 | 16 |
|  | GO:1902531 | 27 | 0.328062 | 1.204494 | 0.095057 | 1 | 13 |
|  | GO:0051716 | 44 | 0.430065 | 1.191079 | 0.111546 | 1 | 31 |
|  | GO:1901605 | 21 | 0.335738 | 1.178763 | 0.218367 | 1 | 9 |
|  | GO:0051726 | 33 | 0.560228 | 1.145522 | 0.084149 | 1 | 28 |
|  | GO:0007017 | 37 | 0.472441 | 1.141973 | 0.209393 | 1 | 26 |
|  | GO:1903047 | 27 | 0.681263 | 1.131134 | 0 | 1 | 26 |
|  | GO:0033554 | 33 | 0.469234 | 1.120802 | 0.111546 | 1 | 24 |
|  | GO:0022402 | 39 | 0.606701 | 1.107205 | 0 | 1 | 35 |
|  | GO:0006508 | 20 | 0.555567 | 1.10586 | 0.287671 | 1 | 17 |
|  | GO:0042221 | 26 | 0.289603 | 1.08453 | 0.28719 | 1 | 10 |
|  | GO:0006974 | 26 | 0.511149 | 1.077573 | 0.097847 | 1 | 20 |
|  | GO:0051603 | 17 | 0.579305 | 1.075567 | 0.181996 | 1 | 15 |
|  | GO:0006520 | 30 | 0.39322 | 1.074909 | 0.203523 | 1 | 15 |
|  | GO:0006281 | 23 | 0.49052 | 1.057216 | 0.195695 | 1 | 18 |
|  | GO:0051347 | 40 | 0.498208 | 1.054749 | 0.28626 | 1 | 38 |
|  | GO:0051641 | 37 | 0.462766 | 1.054028 | 0.315068 | 1 | 26 |
|  | GO:0044265 | 15 | 0.562712 | 1.048184 | 0.181996 | 1 | 13 |
|  | GO:0031099 | 19 | 0.266347 | 1.042345 | 0.298 | 1 | 9 |
|  | GO:0051276 | 29 | 0.535416 | 1.03829 | 0.170254 | 1 | 22 |
|  | GO:0046434 | 15 | -0.49107 | -1.56953 | 0 | 1 | 8 |
|  | GO:0032787 | 21 | -0.39318 | -1.46177 | 0 | 1 | 8 |
|  | GO:0046700 | 15 | -0.38687 | -1.43487 | 0 | 1 | 6 |
|  | GO:0009150 | 25 | -0.43645 | -1.42611 | 0 | 1 | 11 |
|  | GO:0072521 | 25 | -0.43645 | -1.42611 | 0 | 1 | 11 |
|  | GO:0006163 | 25 | -0.43645 | -1.42611 | 0 | 1 | 11 |
|  | GO:1901361 | 19 | -0.42063 | -1.40543 | 0.119772 | 1 | 8 |
|  | GO:0016310 | 15 | -0.40628 | -1.39216 | 0 | 1 | 6 |

**Table S5** The results of GO terms after GSEA analysis in a group C vs. A.

ES: enrichment score, NES: normalized enrichment score, *P*-value: nominal P value, FDR: false discovery rate.

| **GO Term** | **gene set name** | **Size** | **ES** | **NES** | ***P*-value** | ***P*-adjust** | **Leading edge** |
| --- | --- | --- | --- | --- | --- | --- | --- |
| Biological Processes | GO:0099081 | 26 | 0.410363 | 1.198633 | 0.209205 | 0.435952 | 18 |
|  | GO:0044427 | 35 | 0.541303 | 1.001108 | 0.421053 | 0.535901 | 32 |
|  | GO:0099080 | 26 | 0.410363 | 1.198633 | 0.209205 | 0.54494 | 18 |
|  | GO:0043232 | 30 | 0.353125 | 1.018848 | 0.501053 | 0.559868 | 13 |
|  | GO:0043228 | 30 | 0.353125 | 1.018848 | 0.501053 | 0.63985 | 13 |
|  | GO:0044430 | 43 | 0.303732 | 1.054581 | 0.399582 | 0.686947 | 24 |
|  | GO:0099513 | 26 | 0.410363 | 1.198633 | 0.209205 | 0.726586 | 18 |
|  | GO:0005623 | 28 | -0.17231 | -0.54007 | 0.902439 | 0.968643 | 12 |
|  | GO:0044432 | 21 | 0.158365 | 0.459818 | 0.903421 | 0.98875 | 11 |
|  | GO:0005874 | 17 | 0.517609 | 1.286086 | 0.206186 | 1 | 13 |
|  | GO:0099512 | 26 | 0.410363 | 1.198633 | 0.209205 | 1 | 18 |
|  | GO:0005789 | 17 | 0.190709 | 0.596352 | 0.794521 | 1 | 9 |
|  | GO:0000139 | 17 | 0.168853 | 0.516189 | 0.905512 | 1 | 8 |
|  | GO:0044429 | 18 | -0.42037 | -0.98355 | 0.572895 | 1 | 14 |
|  | GO:0005615 | 19 | -0.35022 | -0.89614 | 0.496894 | 1 | 16 |
|  | GO:0044421 | 25 | -0.31634 | -0.83104 | 0.716942 | 1 | 17 |
|  | GO:0098797 | 19 | -0.21421 | -0.80736 | 0.684647 | 1 | 5 |
|  | GO:0030054 | 17 | -0.2146 | -0.72128 | 0.619141 | 1 | 6 |
|  | GO:0031226 | 15 | -0.28549 | -0.71708 | 0.900826 | 1 | 9 |
|  | GO:1902494 | 16 | -0.21535 | -0.61784 | 0.796258 | 1 | 14 |
|  | GO:0098588 | 25 | -0.18013 | -0.61366 | 0.902637 | 1 | 8 |
|  | GO:0031090 | 32 | -0.15807 | -0.59938 | 0.793103 | 1 | 15 |
|  | GO:0044431 | 24 | -0.16004 | -0.55019 | 0.799591 | 1 | 23 |
| Cellular Components | GO:0098797 | 33 | -0.41532 | -1.19362 | 0.203666 | 0.502166 | 17 |
|  | GO:0005615 | 26 | -0.36763 | -1.01502 | 0.503055 | 0.518815 | 15 |
|  | GO:0044449 | 18 | -0.65565 | -1.28343 | 0 | 0.559201 | 14 |
|  | GO:0044429 | 20 | -0.32207 | -1.0391 | 0.40081 | 0.607499 | 8 |
|  | GO:0044421 | 35 | -0.2994 | -0.89379 | 0.492872 | 0.640822 | 18 |
|  | GO:0031090 | 39 | 0.285709 | 0.938717 | 0.388889 | 0.672464 | 25 |
|  | GO:0044432 | 44 | 0.361326 | 0.979688 | 0.388889 | 0.6768 | 30 |
|  | GO:0032993 | 17 | 0.592445 | 0.939297 | 0.606996 | 0.705121 | 13 |
|  | GO:0044815 | 19 | 0.612364 | 0.981139 | 0.606996 | 0.713552 | 15 |
|  | GO:0000139 | 29 | 0.415375 | 0.982702 | 0.495885 | 0.743524 | 22 |
|  | GO:0005911 | 15 | 0.346722 | 1.000028 | 0.399168 | 0.76957 | 8 |
|  | GO:0098588 | 34 | 0.375919 | 1.003063 | 0.286008 | 0.816625 | 24 |
|  | GO:0044431 | 34 | 0.37487 | 1.02203 | 0.498965 | 0.852844 | 24 |
|  | GO:0005623 | 24 | 0.336262 | 1.009591 | 0.484064 | 0.856509 | 15 |
|  | GO:0005789 | 34 | 0.342444 | 1.027674 | 0.388889 | 0.897773 | 19 |
|  | GO:0043232 | 40 | 0.383137 | 1.165671 | 0.195473 | 0.919189 | 22 |
|  | GO:0044427 | 42 | 0.634913 | 1.038881 | 0.205761 | 0.948089 | 35 |
|  | GO:0099513 | 35 | 0.411375 | 1.257089 | 0.201663 | 1 | 21 |
|  | GO:0099512 | 36 | 0.400144 | 1.199242 | 0.201663 | 1 | 21 |
|  | GO:0099080 | 36 | 0.400144 | 1.199242 | 0.201663 | 1 | 21 |
|  | GO:0099081 | 36 | 0.400144 | 1.199242 | 0.201663 | 1 | 21 |
|  | GO:0005874 | 20 | 0.571382 | 1.186595 | 0.2079 | 1 | 15 |
|  | GO:0043228 | 40 | 0.383137 | 1.165671 | 0.195473 | 1 | 22 |
|  | GO:0044430 | 60 | 0.265088 | 1.072533 | 0.202929 | 1 | 29 |
|  | GO:1902494 | 30 | 0.350814 | 1.044204 | 0.388889 | 1 | 18 |
| Molecular Function | GO:0050662 | 21 | -0.32991 | -0.86314 | 0.612524 | 0.808185 | 11 |
|  | GO:0004857 | 20 | -0.31527 | -0.83299 | 0.679089 | 0.818142 | 14 |
|  | GO:0016782 | 15 | -0.29064 | -0.88036 | 0.581781 | 0.824876 | 5 |
|  | GO:0046873 | 30 | -0.40585 | -0.90136 | 0.624016 | 0.835405 | 23 |
|  | GO:0022803 | 23 | -0.30273 | -0.91446 | 0.51751 | 0.877323 | 6 |
|  | GO:0008168 | 19 | -0.23876 | -0.76288 | 0.603113 | 0.899819 | 9 |
|  | GO:0015267 | 23 | -0.30273 | -0.91446 | 0.51751 | 0.939989 | 6 |
|  | GO:0004674 | 21 | -0.2749 | -0.9606 | 0.599613 | 0.9655 | 8 |
|  | GO:0003779 | 24 | -0.42291 | -0.92736 | 0.435897 | 0.988627 | 19 |
|  | GO:0008017 | 18 | 0.5379 | 1.398035 | 0 | 1 | 14 |
|  | GO:0015631 | 19 | 0.507478 | 1.304431 | 0.106212 | 1 | 14 |
|  | GO:0008234 | 21 | 0.385865 | 1.195288 | 0.21611 | 1 | 8 |
|  | GO:0003774 | 20 | 0.371719 | 1.077849 | 0.405063 | 1 | 12 |
|  | GO:0004518 | 16 | 0.327575 | 0.983646 | 0.387795 | 1 | 5 |
|  | GO:0004252 | 30 | 0.290356 | 0.835539 | 0.704365 | 1 | 6 |
|  | GO:0030246 | 27 | 0.210111 | 0.786607 | 0.645669 | 1 | 10 |
|  | GO:0017171 | 36 | 0.235213 | 0.764299 | 0.59332 | 1 | 5 |
|  | GO:0008236 | 36 | 0.235213 | 0.764299 | 0.59332 | 1 | 5 |
|  | GO:0016887 | 19 | 0.236158 | 0.709004 | 0.560825 | 1 | 6 |
|  | GO:0016757 | 35 | 0.216953 | 0.686578 | 0.809055 | 1 | 17 |
|  | GO:0042623 | 16 | 0.237268 | 0.661248 | 0.809623 | 1 | 5 |
|  | GO:0016853 | 17 | 0.189957 | 0.639951 | 0.801255 | 1 | 10 |
|  | GO:0001664 | 17 | 0.213408 | 0.576738 | 0.913127 | 1 | 10 |
|  | GO:0003924 | 25 | 0.14929 | 0.574944 | 0.911243 | 1 | 24 |
|  | GO:0005198 | 33 | 0.145541 | 0.554486 | 0.907025 | 1 | 11 |
|  | GO:0005125 | 16 | 0.172251 | 0.463542 | 0.913127 | 1 | 9 |
|  | GO:0016758 | 16 | 0.171283 | 0.461315 | 0.911591 | 1 | 12 |
|  | GO:0042379 | 15 | 0.150971 | 0.408646 | 0.913127 | 1 | 14 |
|  | GO:0008009 | 15 | 0.150971 | 0.408646 | 0.913127 | 1 | 14 |
|  | GO:0020037 | 16 | -0.5558 | -1.48768 | 0 | 1 | 8 |
|  | GO:0046906 | 17 | -0.5242 | -1.40344 | 0.095331 | 1 | 8 |
|  | GO:0016791 | 16 | -0.45051 | -1.30539 | 0.102564 | 1 | 9 |
|  | GO:0042578 | 23 | -0.42778 | -1.23114 | 0.220907 | 1 | 15 |
|  | GO:0005506 | 24 | -0.41816 | -1.2008 | 0.196498 | 1 | 11 |
|  | GO:0015291 | 23 | -0.47989 | -1.13209 | 0.315789 | 1 | 16 |
|  | GO:0022838 | 20 | -0.37079 | -1.03204 | 0.405512 | 1 | 6 |
|  | GO:0005216 | 20 | -0.37079 | -1.03204 | 0.405512 | 1 | 6 |
|  | GO:0004497 | 16 | -0.38812 | -1.01854 | 0.527237 | 1 | 6 |
|  | GO:0022804 | 33 | -0.42319 | -0.98901 | 0.632094 | 1 | 23 |
|  | GO:0015077 | 24 | -0.4409 | -0.96883 | 0.505906 | 1 | 16 |

**Table S6** The results of GO terms after GSEA analysis in a group C vs. SA.

ES: enrichment score, NES: normalized enrichment score, *P*-value: nominal P value, FDR: false discovery rate.

| **GO Term** | **Gene set name** | **Size** | **ES** | **NES** | ***P*-value** | ***P*-adjust** | **Leading edge** |
| --- | --- | --- | --- | --- | --- | --- | --- |
| Biological Processes | GO:0034622 | 20 | 0.480882 | 0.984719 | 0.39759 | 0.914002 | 17 |
|  | GO:0006974 | 27 | 0.518073 | 1.007817 | 0.514056 | 0.930964 | 24 |
|  | GO:0043413 | 16 | 0.379052 | 0.967369 | 0.491968 | 0.934152 | 10 |
|  | GO:0010941 | 26 | 0.292415 | 1.150527 | 0.186508 | 0.93661 | 11 |
|  | GO:0070925 | 18 | 0.31713 | 0.990118 | 0.469062 | 0.941827 | 11 |
|  | GO:0034645 | 38 | 0.373171 | 1.01505 | 0.490079 | 0.962669 | 30 |
|  | GO:0043067 | 25 | 0.321984 | 1.166323 | 0.186508 | 0.976662 | 11 |
|  | GO:1903047 | 26 | 0.743731 | 1.327759 | 0 | 1 | 24 |
|  | GO:0010564 | 17 | 0.604381 | 1.308852 | 0 | 1 | 15 |
|  | GO:0007017 | 28 | 0.532935 | 1.303815 | 0.103792 | 1 | 21 |
|  | GO:0007346 | 15 | 0.684119 | 1.300081 | 0 | 1 | 14 |
|  | GO:0022402 | 35 | 0.682114 | 1.291943 | 0 | 1 | 30 |
|  | GO:0051276 | 25 | 0.69338 | 1.214418 | 0 | 1 | 20 |
|  | GO:0051726 | 32 | 0.559384 | 1.20015 | 0 | 1 | 26 |
|  | GO:0042981 | 25 | 0.321984 | 1.166323 | 0.186508 | 1 | 11 |
|  | GO:0033554 | 31 | 0.407921 | 1.048513 | 0.37751 | 1 | 24 |
|  | GO:0006260 | 24 | 0.565701 | 1.026916 | 0.299197 | 1 | 23 |
|  | GO:0006281 | 22 | 0.55803 | 1.025477 | 0.295181 | 1 | 22 |
|  | GO:0051716 | 41 | 0.291519 | 1.023892 | 0.383534 | 1 | 16 |
|  | GO:0009059 | 38 | 0.373171 | 1.01505 | 0.490079 | 1 | 30 |
|  | GO:0007155 | 28 | -0.4146 | -1.29844 | 0.104628 | 1 | 11 |
|  | GO:0022610 | 28 | -0.4146 | -1.29844 | 0.104628 | 1 | 11 |
|  | GO:0009892 | 15 | -0.35807 | -1.22845 | 0.108871 | 1 | 7 |
|  | GO:0030029 | 15 | -0.43972 | -1.11131 | 0.315261 | 1 | 8 |
|  | GO:1901575 | 41 | -0.39113 | -1.10437 | 0.409639 | 1 | 28 |
|  | GO:0033043 | 25 | -0.30363 | -1.09458 | 0.296371 | 1 | 8 |
|  | GO:0017144 | 21 | -0.44768 | -1.08215 | 0.291165 | 1 | 16 |
|  | GO:0048585 | 17 | -0.42336 | -1.08202 | 0.295181 | 1 | 10 |
|  | GO:0044248 | 40 | -0.37705 | -1.07749 | 0.409639 | 1 | 27 |
|  | GO:0006811 | 20 | -0.49674 | -1.07557 | 0.381526 | 1 | 15 |
|  | GO:1901361 | 17 | -0.42377 | -1.03689 | 0.533469 | 1 | 12 |
|  | GO:0051246 | 17 | -0.27177 | -1.02838 | 0.335366 | 1 | 5 |
|  | GO:0032268 | 17 | -0.27177 | -1.02838 | 0.335366 | 1 | 5 |
|  | GO:0006082 | 46 | -0.32896 | -1.02469 | 0.376238 | 1 | 24 |
|  | GO:1901605 | 17 | -0.39976 | -1.0003 | 0.600402 | 1 | 10 |
|  | GO:0030030 | 16 | -0.45609 | -0.99827 | 0.379518 | 1 | 13 |
|  | GO:0043436 | 45 | -0.31389 | -0.99494 | 0.376238 | 1 | 23 |
|  | GO:0019439 | 16 | -0.39091 | -0.98886 | 0.626775 | 1 | 11 |
|  | GO:0019752 | 44 | -0.30429 | -0.98759 | 0.376238 | 1 | 22 |
|  | GO:0098771 | 19 | -0.41679 | -0.98656 | 0.504951 | 1 | 15 |
| Cellular Components | GO:0005874 | 22 | -0.46832 | -1.08117 | 0.30426 | 0.740092 | 16 |
|  | GO:0044430 | 50 | -0.29166 | -1.00902 | 0.480808 | 0.760122 | 24 |
|  | GO:0099081 | 33 | -0.37652 | -1.12174 | 0.091278 | 0.761956 | 23 |
|  | GO:0005794 | 18 | -0.36506 | -1.05831 | 0.292929 | 0.800572 | 7 |
|  | GO:0044427 | 45 | -0.54029 | -1.01544 | 0.322515 | 0.825849 | 31 |
|  | GO:0044815 | 15 | -0.53231 | -1.02115 | 0.592292 | 0.865658 | 10 |
|  | GO:0005887 | 20 | 0.365576 | 0.895656 | 0.607438 | 0.88094 | 14 |
|  | GO:1902494 | 23 | 0.166842 | 0.650812 | 0.892644 | 0.9006 | 7 |
|  | GO:0044432 | 28 | -0.20123 | -0.6936 | 0.684631 | 0.908202 | 17 |
|  | GO:0099080 | 33 | -0.37652 | -1.12174 | 0.091278 | 0.914347 | 23 |
|  | GO:0000139 | 15 | -0.28414 | -0.78726 | 0.787018 | 0.951284 | 9 |
|  | GO:0044431 | 21 | -0.21636 | -0.69988 | 0.893069 | 0.955574 | 10 |
|  | GO:0031226 | 22 | 0.252792 | 0.726178 | 0.667335 | 0.963042 | 12 |
|  | GO:0005623 | 22 | -0.14866 | -0.5387 | 0.894325 | 0.964559 | 3 |
|  | GO:0098588 | 20 | -0.24119 | -0.7959 | 0.59596 | 0.972619 | 12 |
|  | GO:0031090 | 24 | -0.21595 | -0.7286 | 0.807453 | 0.983733 | 14 |
|  | GO:0005789 | 22 | -0.20504 | -0.71248 | 0.690802 | 0.984645 | 14 |
|  | GO:0005615 | 20 | 0.374244 | 1.10614 | 0.27821 | 1 | 9 |
|  | GO:0044421 | 30 | 0.318618 | 0.971593 | 0.60124 | 1 | 12 |
|  | GO:0030054 | 18 | 0.286352 | 0.897204 | 0.608519 | 1 | 9 |
|  | GO:0043228 | 39 | -0.40546 | -1.19707 | 0 | 1 | 23 |
|  | GO:0043232 | 39 | -0.40546 | -1.19707 | 0 | 1 | 23 |
|  | GO:0099513 | 32 | -0.38982 | -1.17383 | 0.091278 | 1 | 22 |
|  | GO:0099512 | 33 | -0.37652 | -1.12174 | 0.091278 | 1 | 23 |
|  | GO:0098797 | 25 | -0.22608 | -0.82401 | 0.469388 | 1 | 8 |
| Molecular Function | GO:0001653 | 28 | -0.34518 | -0.99764 | 0.488798 | 0.837532 | 19 |
|  | GO:0042379 | 15 | -0.41762 | -0.92708 | 0.580122 | 0.855583 | 13 |
|  | GO:0016791 | 24 | -0.27068 | -0.93086 | 0.483607 | 0.887845 | 9 |
|  | GO:0005126 | 18 | -0.43774 | -0.94947 | 0.488844 | 0.889008 | 16 |
|  | GO:0008528 | 28 | -0.34518 | -0.99764 | 0.488798 | 0.889878 | 19 |
|  | GO:0022803 | 38 | -0.33528 | -1.01393 | 0.474645 | 0.908005 | 16 |
|  | GO:0015267 | 38 | -0.33528 | -1.01393 | 0.474645 | 0.972863 | 16 |
|  | GO:0022839 | 23 | -0.4507 | -1.28296 | 0.097363 | 1 | 11 |
|  | GO:0005261 | 17 | -0.53358 | -1.23668 | 0.198783 | 1 | 10 |
|  | GO:0022836 | 24 | -0.43403 | -1.23481 | 0.097363 | 1 | 11 |
|  | GO:0022838 | 30 | -0.43562 | -1.18818 | 0.198783 | 1 | 15 |
|  | GO:0005216 | 30 | -0.43562 | -1.18818 | 0.198783 | 1 | 15 |
|  | GO:0004896 | 21 | -0.46569 | -1.15367 | 0 | 1 | 15 |
|  | GO:0061134 | 16 | -0.46905 | -1.12674 | 0.375502 | 1 | 12 |
|  | GO:0001637 | 16 | -0.45323 | -1.11267 | 0.210953 | 1 | 11 |
|  | GO:0004950 | 16 | -0.45323 | -1.11267 | 0.210953 | 1 | 11 |
|  | GO:0003779 | 32 | -0.40704 | -1.08456 | 0.399177 | 1 | 19 |
|  | GO:0030414 | 15 | -0.44529 | -1.06214 | 0.473896 | 1 | 11 |
|  | GO:0046873 | 34 | -0.36863 | -1.04304 | 0.42623 | 1 | 20 |
|  | GO:0015077 | 29 | -0.36622 | -1.02496 | 0.411885 | 1 | 13 |
|  | GO:0017171 | 34 | 0.347138 | 1.246184 | 0.098456 | 1 | 5 |
|  | GO:0008236 | 34 | 0.347138 | 1.246184 | 0.098456 | 1 | 5 |
|  | GO:0015631 | 22 | 0.516635 | 1.228055 | 0.09761 | 1 | 14 |
|  | GO:0008234 | 19 | 0.481591 | 1.211357 | 0.184 | 1 | 10 |
|  | GO:0008017 | 21 | 0.533513 | 1.189579 | 0.219124 | 1 | 14 |
|  | GO:0003924 | 28 | 0.412289 | 1.169077 | 0.196 | 1 | 18 |
|  | GO:0016853 | 24 | 0.519127 | 1.136379 | 0.087221 | 1 | 19 |
|  | GO:0004252 | 29 | 0.317573 | 1.120686 | 0.289575 | 1 | 4 |
|  | GO:0005198 | 49 | 0.335901 | 1.113636 | 0.39243 | 1 | 32 |
|  | GO:0003774 | 23 | 0.43079 | 1.111355 | 0.511952 | 1 | 14 |
|  | GO:0046982 | 19 | 0.570386 | 1.078188 | 0.393509 | 1 | 14 |
|  | GO:0016874 | 21 | 0.405667 | 1.068545 | 0.276 | 1 | 16 |
|  | GO:0004497 | 19 | 0.296579 | 1.043957 | 0.510763 | 1 | 4 |
|  | GO:0008289 | 30 | 0.303625 | 1.038446 | 0.392857 | 1 | 13 |
|  | GO:0016887 | 27 | 0.347726 | 0.995638 | 0.506876 | 1 | 12 |
|  | GO:0055106 | 36 | 0.558521 | 0.986163 | 0.288066 | 1 | 36 |
|  | GO:0097027 | 36 | 0.558521 | 0.986163 | 0.288066 | 1 | 36 |
|  | GO:0010997 | 36 | 0.558521 | 0.986163 | 0.288066 | 1 | 36 |
|  | GO:0140098 | 20 | 0.358365 | 0.956851 | 0.404858 | 1 | 11 |
|  | GO:0008509 | 18 | 0.311953 | 0.948778 | 0.398 | 1 | 4 |
